# Regional Brain Volume Variation Across Adulthood: A Cross-Sectional MRI Analysis of Age, Sex, and Hemispheric Asymmetry

**DOI:** 10.3390/life16081356

**Published:** 2026-08-18

**Authors:** Tanmoy Debnath, Md Geaur Rahman, Sourabhi Debnath, Minh Chau

**Affiliations:** 1 CSU Engineering, Charles Sturt University, Bathurst, NSW 2795, Australia; 2 School of Computing, Mathematics and Engineering, Charles Sturt University, Port Macquarie, NSW 2444, Australia; grahman@csu.edu.au; 3 School of Computing, Mathematics and Engineering, Charles Sturt University, Bathurst, NSW 2795, Australia; sdebnath@csu.edu.au; 4 School of Dentistry and Medical Sciences, Charles Sturt University, Orange, NSW 2800, Australia

**Keywords:** brain ageing, structural MRI, regional brain volume, sex differences, hemispheric asymmetry, intracranial volume correction, subcortical structures, lifespan age association, brain volumetry, AgeRisk cohort

## Abstract

Distinguishing normative cross-sectional structural age differences from early neurodegeneration requires region-specific characterisation of cross-sectional age-related variation, yet findings remain sensitive to modelling strategy and intracranial volume (ICV) correction. T1-weighted MRI from 187 healthy adults (97 female, 90 male; aged 16.22–81.48 years) in the AgeRisk dataset were segmented using Vol2Brain. Multiple regression models applied to 19 predefined brain regions tested linear and quadratic age, sex, ICV, and signal-to-noise ratio (FDR-corrected within predictor families); hemispheric asymmetry indices were Bonferroni-corrected. Three hypothesis domains were preregistered: age associations, sex differences, and hemispheric asymmetry. Negative cross-sectional age associations were observed in subcortical (hippocampus, amygdala, caudate, putamen, and nucleus accumbens) and cortical regions (insula, temporal and occipital lobes; all $p_{\mathrm{FDR}}<0.05$). The parietal lobe showed significant quadratic curvature ($p_{\mathrm{FDR}}=0.008$); total cortical grey matter showed no age effect. White matter exhibited a positive linear rather than the hypothesised inverted-U relationship. After ICV adjustment, females showed greater grey-matter-dominant volumes and males greater cerebrospinal fluid volume (both $p_{\mathrm{FDR}}<0.05$). Sex-by-age interactions in four subcortical regions indicated shallower negative cross-sectional age associations in females (all $p_{\mathrm{FDR}}\leq 0.034$). Age was associated with reduced parietal and temporal hemispheric asymmetry (both p<0.001). Findings confirm regionally heterogeneous ageing, ICV-adjusted sex differences, and a selective negative cross-sectional association between age and hemispheric asymmetry, consistent with the broader lifespan literature.

## 1. Introduction

**Figure 1:**
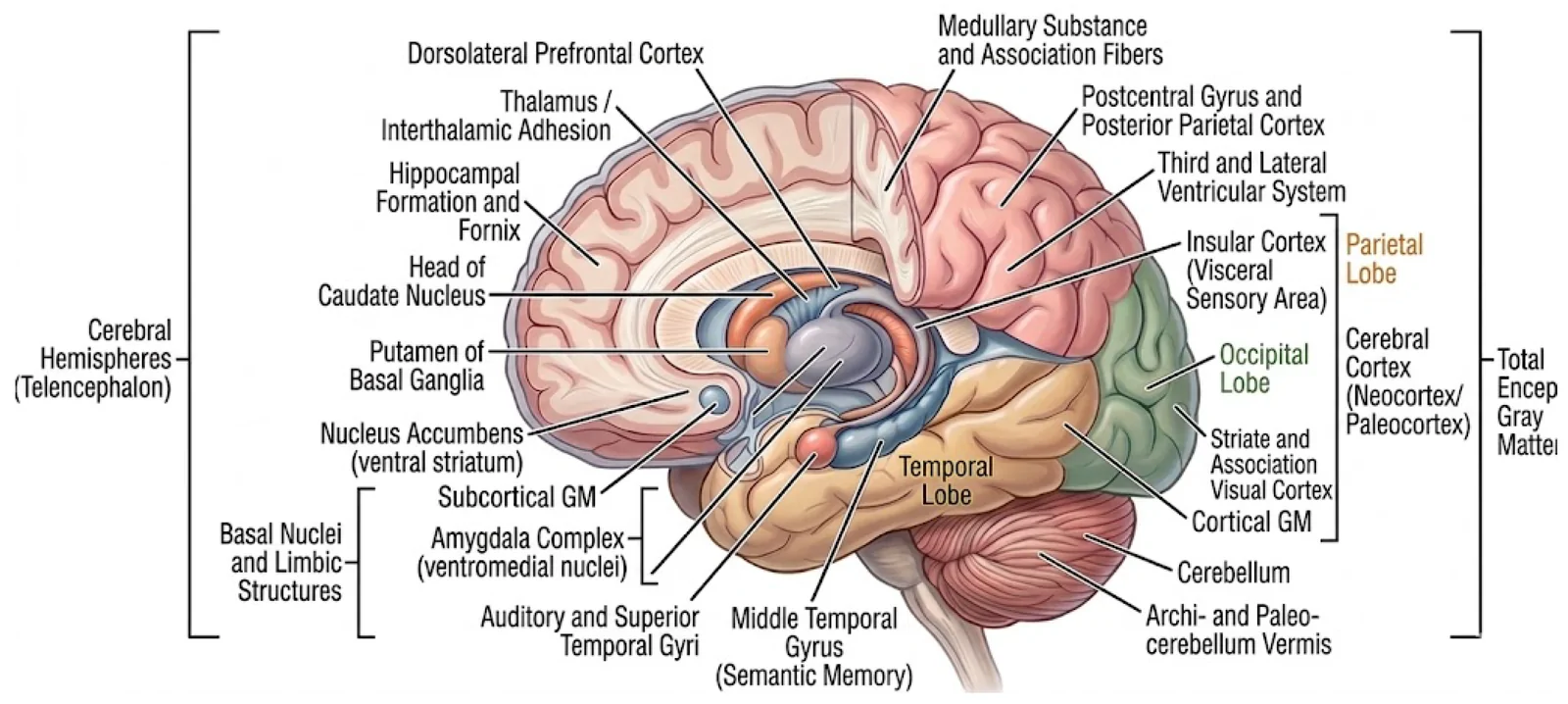
Anatomical representation of the 19 pre-specified regions of interest.

Distinguishing age-related structural variation in the healthy adult brain from the earliest structural signatures of neurodegeneration remains a central challenge in clinical neuroscience [1]. This requires the precise characterisation of region-specific volumetric variation across adulthood, as age-related changes differ substantially in onset, magnitude, and spatial distribution across neuroanatomical systems [2]. Large-scale neuroimaging studies consistently show that cross-sectional structural age differences is heterogeneous rather than global, with subcortical structures such as the hippocampus, amygdala, caudate, and putamen typically showing monotonic decline. However, cortical regions exhibit more variable patterns depending on functional specialisation and developmental timing [3,4,5]. White matter (WM) exhibits nonlinear lifespan profiles in large cohorts, increasing through early and mid-adulthood before declining. However, such dynamics are sometimes undetectable in smaller cross-sectional samples, where observed effects may reflect between-person differences rather than within-person change [6,7,8]. Whether these differences vary with age, particularly within subcortical structures, remains incompletely characterised. We therefore conducted a preregistered analysis of ICV-adjusted sex differences and sex-by-age interactions across 19 brain regions (Figure 1).

Sex differences represent a second major axis of structural brain variation across adulthood, but their interpretation is complicated by systematic differences in ICV between males and females [9]. As males exhibit larger ICV on average, valid inference requires regression-based adjustment to dissociate regional neuroanatomical variation from global head-size scaling [10]. Studies generally report relatively greater GM volumes in females [11] and increased CSF volume in males [12] after correction, although estimates vary across cohorts and segmentation pipelines [13]. Potential explanations for sex-differentiated structural ageing are likely multifactorial [14,15]. Candidate pathways include variation in gonadal-steroid exposure across the life course, sex-related differences in cerebrovascular function and vascular ageing, immune–glial signalling, and metabolic health. These pathways may interact rather than operate independently. In particular, hormonal transitions may influence vascular signalling and vascular remodelling across adulthood, although the direction and magnitude of such effects are likely to depend on age, endocrine stage, and the vascular phenotype assessed [16]. Sex-dependent regulation of glucose transport, glycolysis, and mitochondrial function may contribute to variation in brain metabolic vulnerability with ageing [17]. However, structural MRI alone cannot identify these mechanisms, and the present study was designed to characterise regional volumetric associations rather than to test biological mediation.

Hemispheric asymmetry constitutes a third dimension of structural organisation that may change systematically with age [18]. Established lateralisation patterns are well documented, but evidence on age-related modification remains sparse [19,20,21]. The HAROLD framework proposes reduced functional lateralisation in older adults [22,23], but whether comparable structural patterns exist and can be quantified using continuous volumetric asymmetry indices remains underexplored.

This cross-sectional analysis applies Vol2Brain automated segmentation [24,25] to AgeRisk MRI data [26]. For each of 19 predefined regions, we model linear and quadratic age associations while adjusting for sex, ICV, and image quality, quantified by signal-to-noise ratio (SNR). Hemispheric asymmetry is also quantified using continuous indices across bilateral structures to evaluate age-related variation in lateralisation. The preregistered core analyses, therefore, address three domains (Table 1): age associations, sex differences in regional volume, and hemispheric asymmetry. An additional analysis of sex effects on asymmetry is treated as secondary.

The primary contributions of this work are three-fold. First, it provides a cross-sectional reference analysis of FDR-controlled age associations across 19 age-sensitive brain regions, distinguishing linear decline in subcortical structures from more variable cortical patterns within a single analytic framework. Second, it demonstrates ICV-adjusted sex differences and sex-by-age interactions using a consistent regression-based correction framework. This reveals female-specific age effects with shallower age-related slopes that remain significant after family-wise multiple-testing correction. Third, it presents confirmatory continuous-index structural asymmetry findings across 12 pre-specified bilateral regions, quantifying age-related reductions primarily in posterior cortical structures while clearly separating these results from exploratory extensions. These contributions integrate three understudied dimensions of structural brain organisation: regional age variation, ICV-sensitive sexual dimorphism, and hemispheric lateralisation. The framework is implemented within a disciplined, preregistered design and it explicitly distinguishes cross-sectional associations from longitudinal inference.

The remainder of this paper is structured as follows. Section 2 describes the methodology. Section 3 presents the results and discussion for age associations, sex differences, and hemispheric asymmetry, with detailed coefficients provided in the Supplementary Materials. Section 4 outlines the limitations and future directions. Section 5 provides a concise conclusion, including a summary of the hypothesis outcomes.

## 2. Methods

### 2.1. Participants and Study Design

This study is a secondary analysis based on previously published materials and de-identified structural MRI data obtained from the open-access AgeRisk dataset [26,27]. No new participant recruitment, data collection, or intervention was undertaken for the present analysis. Cross-sectional T1-weighted MRI data from the AgeRisk dataset comprised 187 healthy right-handed adults (97 female, 90 male) aged 16.22–81.48 years (M=45.05, SD=19.27) following exclusions for scanner refusal (n=2), withdrawal prior to MRI (n=11), neurological or psychiatric diagnoses, MRI contraindications, and non-right-handedness [27]. Age distributions did not differ significantly between sexes (females: M=44.4 years; males: M=45.9 years; t(185)=0.52, p=0.605), whereas males exhibited substantially greater intracranial volume (ICV) than females ($1384.9\pm 124.7\;{\mathrm{cm}}^{3}$ versus $1254.4\pm 130.1\;{\mathrm{cm}}^{3}$; t(185)=6.99, p<0.001, d=0.91), representing an approximately 10.4% difference consistent with the prior literature [28,29,30]. The original AgeRisk study procedures were approved by the University Hospital Basel ethics committee and conducted in accordance with the Declaration of Helsinki.

### 2.2. MRI Acquisition and Preprocessing

Structural T1-weighted images were acquired on a Siemens MAGNETOM Prisma 3T scanner. Full acquisition parameters are documented in the BIDS JSON sidecars (OpenNeuro) and the OSF protocol [31]. All participants were scanned on the same scanner.

Images were skull-stripped using FSL BET with a fractional intensity threshold of f=0.5. One participant (sub-138) required an adapted threshold (f=0.4) because of preprocessing artefacts; the resulting ICV was within the normal range ($1554.9\;{\mathrm{cm}}^{3}$, z=1.66). All skull-stripped images were visually inspected before segmentation.

### 2.3. Vol2Brain Segmentation

Skull-stripped T1-weighted images were processed using the Vol2Brain automated segmentation pipeline. The pipeline incorporates non-local means denoising, N4 bias field correction, affine registration to MNI space, tissue classification, and multi-atlas label propagation with majority voting [32]. Automated segmentation frameworks of this type are increasingly used in quantitative MRI workflows because they enable scalable and reproducible volumetric analysis across large datasets [33,34]. This yielded absolute volumes (${\mathrm{cm}}^{3}$) for 135 neuroanatomical structures, which were aggregated into 19 regions of interest selected *a priori* for age sensitivity and theoretical relevance (Supplementary Table S1).

Two classes of ROI arise from this pipeline and differ in tissue composition. Global compartments (total GM, cortical GM, subcortical GM, cerebellar GM, total WM, and CSF) derive from Vol2Brain’s voxel-wise tissue classification and are tissue-pure by construction. The cortical lobes used here (frontal, temporal, parietal, and occipital) instead derive from multi-atlas label propagation using an augmented Neuromorphometrics protocol [25], in which each lobe is the sum of its constituent cortical gyri (e.g., the frontal lobe comprises FRP, GRe, OpIFG, OrIFG, TrIFG, MFC, MFG, AOrG, LOrG, MOrG, POrG, PrG, MPrG, SCA, SFG, MSFG, and SMC).

We verified the tissue composition of these lobar ROIs arithmetically in the full analytic sample (n=187). First, cerebral white matter is returned by this pipeline only as a single pooled compartment (*Cerebrum WM*); Second, the frontal lobe total equals the sum of its 17 constituent gyri in every participant to within 0.03 cm^3^ (mean absolute discrepancy 0.006% of frontal volume), consistent with rounding across 17 already-rounded values rather than an omitted tissue component (Table 2 gives a full worked example for two participants selected at random). Third, the frontal lobe, together with the temporal, parietal, occipital, insular, and limbic lobes reproduces the pipeline’s independently computed cortical GM volume to within 0.02 cm^3^ per participant (mean absolute discrepancy 0.001%). Fourth, as a control, deliberately adding whole-brain WM to this six-lobe sum before comparing it against cortical GM increases the mean discrepancy to 394.5 cm^3^, confirming that the close match above reflects the absence of a white-matter component rather than coincidental cancellation. Frontal lobe volume, as used throughout the analyses below, is therefore a verified component of cortical GM rather than a GM/WM composite, and the same holds for the temporal, parietal, and occipital ROIs.

Vol2Brain demonstrates strong test–retest reliability and agreement with FreeSurfer for key subcortical regions. Total ICV and scan-level SNR are extracted directly from Vol2Brain outputs as covariates.

Thalamic estimates show high variance in absolute volume (CV > 40%; left = 51.0%, right = 56.9%, total = 53.4%), consistent with known Vol2Brain instability for this structure; absolute thalamic volume is therefore reported in Supplementary Table S1 only and excluded from the primary volumetric inferences (H1, H2). This exclusion does not automatically extend to the thalamic asymmetry index (AI), as it is a within-subject ratio of paired left- and right-hemisphere estimates rather than an absolute magnitude, and is therefore differentially sensitive to shared versus hemisphere-specific sources of segmentation noise (i.e., correlated bilateral scaling error is cancelled). Empirically, the thalamic AI exhibits excellent statistical stability (SD = 11.38%), with lower variance than other included subcortical structures (e.g., hippocampus SD = 19.37%, pallidum SD = 22.67%). Therefore, the thalamus is retained in the confirmatory asymmetry family.

### 2.4. Hypothesis-Specific Analyses: Volume Models

#### 2.4.1. H1: Age Associations (19 Regions)

Following established lifespan modelling approaches, 19 regional brain volumes are modelled as a function of linear and quadratic age effects to capture potential nonlinear associations. Sex and total ICV are included as continuous covariates in an ANCOVA framework, rather than as a ratio-based correction, to avoid bias arising from non-zero intercepts in the ICV–volume relationship [35,36]. This covariate-based approach converges with recent methodological evidence that intracranial volume normalisation accounts for the great majority of apparent sex differences in regional brain volume, underscoring the importance of correction-method choice when interpreting ICV-adjusted sex effects [37]. Scanner SNR is included to control for motion-related partial-volume effects, as age-related head motion can systematically underestimate GM if uncontrolled [38].

The decision to model age using linear and quadratic terms, rather than penalised regression splines or generalised additive models (GAMs/GAMLSS), reflects three considerations. First, H1a-ii and H1b are confirmatory, pre-registered hypotheses framed around a fixed coefficient ($\beta_{2}$). In contrast, GAM/GAMLSS smooth terms are evaluated using approximate Wald-type tests conditional on an estimated smoothing parameter, which neglect smoothing-parameter uncertainty [39] and therefore constitute a different inferential target from the pre-specified coefficient-based test. Adopting a data-driven smoother post hoc would have converted a confirmatory test into an exploratory one. Second, flexible nonlinear smoothing is comparatively data-hungry. Normative GAMLSS lifespan brain-volume charts have been derived from aggregated cross-sectional samples far larger than the present cohort (101,457 individuals; [2]), reflecting the larger effective sample sizes generally required to estimate smooth curvature without overfitting. Third, the oldest age group is comparatively sparse (n=25 for age ≥70 years), where non-parametric smooths are most susceptible to boundary variance. Consequently, an unconstrained smoother is not necessarily preferable to a globally constrained quadratic model in the age range where late-life nonlinearity is most likely. We treat this as a bias–variance trade-off appropriate to the present sample size rather than a settled question and assess its consequences empirically where feasible (Section 3.2.4).

For each region, the following model is fitted [40,41,42]:

(1) $${\mathrm{Volume}}_{i}=\beta_{0}+\beta_{1}{\mathrm{Age}}_{i}+\beta_{2}{\mathrm{Age}}_{i}^{2}+\beta_{3}{\mathrm{Sex}}_{i}+\beta_{4}{\mathrm{ICV}}_{i}+\beta_{5}{\mathrm{SNR}}_{i}+\varepsilon_{i}$$

where ${\mathrm{Age}}_{i}$ is mean-centred (M=45.05 years), ${\mathrm{Sex}}_{i}$ is dummy-coded as male (=1), female (=2), and $\varepsilon_{i}$ denotes the error term capturing unexplained variation, including measurement error, omitted variables, and random noise. ${\mathrm{ICV}}_{i}$ and ${\mathrm{SNR}}_{i}$ are left uncentred because their absolute scales are interpretable. Unstandardised coefficients (β) have units of ${\mathrm{cm}}^{3}/\mathrm{year}$ for linear age, ${\mathrm{cm}}^{3}/{\mathrm{year}}^{2}$ for quadratic age, and ${\mathrm{cm}}^{3}$ for ${\mathrm{Sex}}_{i}$, ${\mathrm{ICV}}_{i}$, and ${\mathrm{SNR}}_{i}$. Standardised beta coefficients are additionally computed for each predictor to facilitate comparison of effect magnitudes across regions; these coefficients are unitless and reflect effects expressed in standard deviation units. Positive $\beta_{3}$ indicates greater volume in females relative to males at mean-centred age and at reference ${\mathrm{ICV}}_{i}$ and ${\mathrm{SNR}}_{i}$ values of 0. Coefficient $\beta_{4}$ quantifies head-size scaling. All 19 ${\mathrm{SNR}}_{i}$ coefficients are non-significant after FDR correction (all $p_{\mathrm{FDR}}>0.57$), indicating that image quality does not systematically bias the estimates.

For each predictor family, *p*-values across the 19 regions are submitted to Benjamini–Hochberg false discovery rate (FDR) correction at α=0.05 (m=19). Correction is applied separately within each predictor family rather than across all predictors simultaneously. This strategy follows the principle that each predictor family constitutes a distinct inferential domain addressing a separate scientific question. Effect sizes are reported as semi-partial $R^{2}$ ($pR^{2}$), representing the unique variance explained by each predictor after accounting for all others; 0.05 is interpreted as moderate and 0.10 as large. Statistical significance is reported as $p_{\mathrm{FDR}}<0.05$, $p_{\mathrm{FDR}}<0.01$, and $p_{\mathrm{FDR}}<0.001$. Model fit is reported as adjusted $R^{2}$ ($R_{\mathrm{adj}}^{2}$).

The preregistered H1 analyses are as follows:

- H1a-i: Linear age effect ($\beta_{1}$) across 9 subcortical regions (FDR, m=19).
- H1a-ii: Quadratic age effect ($\beta_{2}$) in the parietal lobe (FDR).
- H1b: Quadratic age effect ($\beta_{2}$) in WM (FDR).
- H1c: Rank ordering of linear age $pR^{2}$, with insula and striatum expected to be highest.

Where the quadratic term ($\beta_{2}$) was FDR-significant, vertex age was calculated as

(2) $$t_{\mathrm{peak}}=-\frac{\beta_{1}}{2\beta_{2}}+45.05$$

As vertex estimates from polynomial models can be sensitive to specification, these values are interpreted descriptively rather than as precise biological transition points.

#### 2.4.2. H2: Sex Differences (19 Regions)

Sex main effects are evaluated using the $\beta_{3}$ coefficient across all 19 regions, with FDR correction applied at m=19. The preregistered H2 analyses are as follows:

- H2a: Sex main effects in GM regions.
- H2b: Sex main effects in CSF.
- H2c: Sex main effects in cerebellum and WM, where null effects are expected.
- H2d: Sex × age interaction terms tested across all 19 regions as a sixth predictor family (FDR, m=19).

#### 2.4.3. H3: Hemispheric Asymmetry (Confirmatory: 12 Regions)

For 12 bilateral structures (hippocampus, amygdala, caudate, putamen, pallidum, thalamus, nucleus accumbens, insula, frontal lobe, parietal lobe, temporal lobe, and cerebrum), asymmetry indices (AI) are computed as

(3) $$\mathrm{AI}=\left[\frac{R-L}{(R+L)/2}\right]\times 100$$

where *R* and *L* are right and left hemisphere volumes (${\mathrm{cm}}^{3}$), respectively. Positive AI values indicate rightward asymmetry, whereas negative values indicate leftward asymmetry. Segmentation failures (hemivolume $=0.0\;{\mathrm{cm}}^{3}$; n=1–7 cases, <4%) or implausible values (|AI|>100) are treated as missing.

The thalamus is retained in this confirmatory asymmetry analysis despite its exclusion from the primary volumetric inferences (H1, H2), and this asymmetric treatment is justified on statistical grounds specific to the properties of a ratio-based index. First, left and right thalamic volumes are very highly correlated across participants (r=0.973, n=187), indicating that the majority of the coefficient-of-variation instability documented for absolute thalamic volume reflects a shared, scan-level source of variance common to both hemispheres, rather than independent left/right segmentation error. Because AI is computed as a normalised right–left difference, noise sources common to both hemispheres are attenuated in AI relative to their effect on either hemisphere volume considered alone. Second, thalamic AI shows only a weak association with absolute thalamic volume (r=0.135, t(185)=1.85, p=0.066) and is uncorrelated with scan-level SNR (r=−0.008, p=0.91), indicating that AI does not simply re-express the volumetric instability documented for the absolute measure. We note that the coefficient of variation is not an appropriate reliability metric for AI itself: AI is a signed index distributed around a small, near-zero mean, so CV is highly sensitive to the denominator and does not meaningfully quantify measurement instability for this class of variable. We therefore assess the reliability of thalamic AI via its association with volume and SNR rather than via its own CV. Hence, thalamic AI is treated as a distinct derived measure with different noise properties from thalamic volume and is reported with the same confidence as the other 11 confirmatory regions.

Population-level asymmetry (H3a) is tested using one-sample *t*-tests or Wilcoxon signed-rank tests against zero, with a uniform Bonferroni-corrected threshold for 12 tests (α=0.05/12=0.004, m=12). Age–AI associations (H3b) are tested across the confirmatory regions (m=12) using partial Pearson correlations controlling for sex and ICV, with Bonferroni correction at α=0.05/12=0.004. A subsidiary methodological check using ordinary least squares regression evaluates the inclusion of a quadratic age term to assess whether structural dedifferentiation is adequately characterised as a linear association.

Three additional bilateral regions (cerebellum, occipital lobe, and WM) are examined in an exploratory extension and are reported separately. All analyses are conducted in Python 3.12 using NumPy 2.1 [43], SciPy 1.14 [44], and pandas 2.2 [45]. No data imputation is performed [46].

As the present study employed a cross-sectional design, all reported age effects represent differences between individuals of different ages rather than within-individual longitudinal changes. Hence, the findings are interpreted as cross-sectional age–volume associations rather than biological ageing associations. Longitudinal studies are required to distinguish true ageing effects from potential cohort differences.

## 3. Results and Discussion

### 3.1. Sample Characteristics

**Figure 2:**
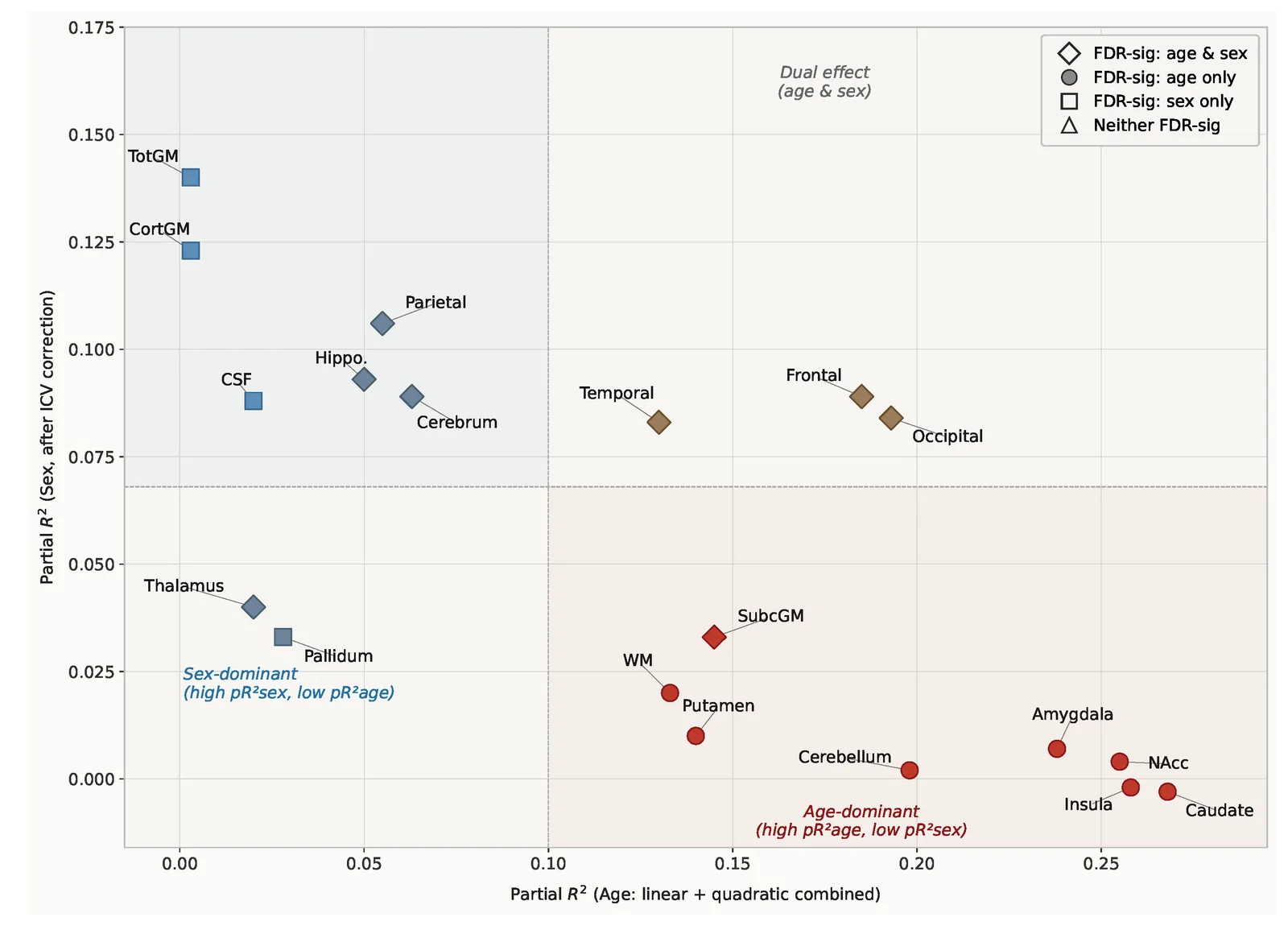
Age- versus sex-sensitivity of regional brain volumes. Symbols indicate regions with significant dual (diamonds), sex-only (squares), or age-only (circles) effects after FDR correction. Shaded areas highlight sex-dominant (blue) and age-dominant (pink) regions based on partial effect sizes ($pR^{2}$).

Table 3 presents decade-stratified mean volumes (SD) for the 19 brain regions, stratified by sex. Table 4 summarises ICV-adjusted sex differences across regions, highlighting FDR-significant predictors. Full coefficients are provided in Supplementary Table S1, showing variation in $pR^{2}$ across regions and predictor domains. Regional differences in age- and sex-sensitivity are summarised in Figure 2, which plots the partial $R^{2}$ for age (linear and quadratic combined) against the partial $R^{2}$ for sex across 19 brain regions. SNR shows no significant associations with regional volumes after FDR correction across all 19 regions, indicating that image quality does not systematically bias volumetric estimates.

### 3.2. Results: Age-Related Regional Brain Volume Associations

#### 3.2.1. Subcortical Structures (H1a-i)

**Figure 3:**
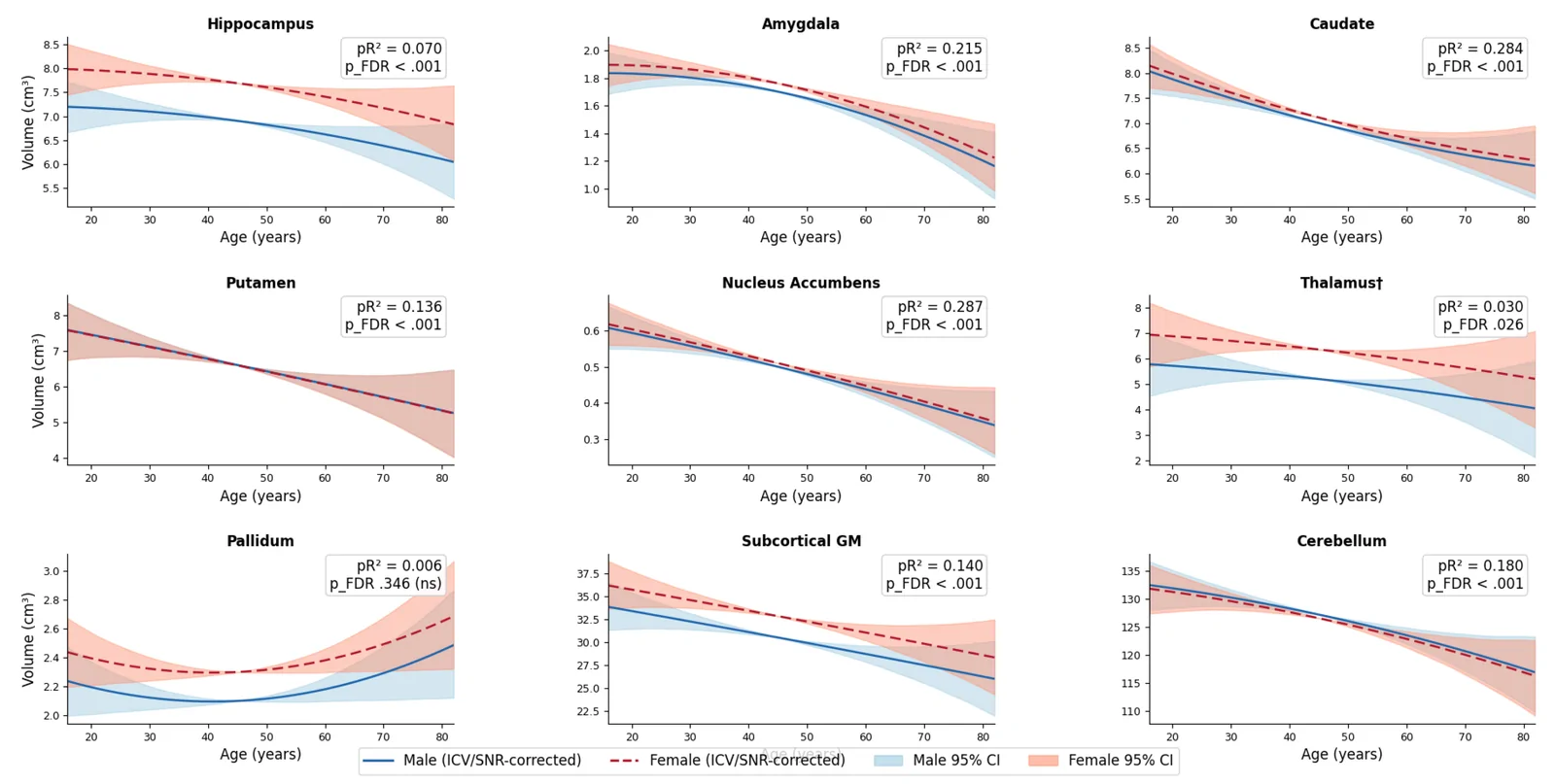
ICV- and SNR-corrected cross-sectional age profiles across nine subcortical regions. Sex-specific quadratic polynomial fits are shown for males (blue) and females (red), and shaded bands represent bootstrapped 95% confidence intervals (CIs). Results directly support hypothesis H1a-i. The thalamic absolute-volume trend is shown for completeness but is excluded from confirmatory volumetric inference because of high segmentation variance (see Section 3.4 for the distinct treatment of thalamic hemispheric asymmetry).

Age associations for nine subcortical regions, corrected for ICV and SNR, are presented in Figure 3, with quadratic polynomial fits and bootstrapped 95% CIs shown separately for males and females. Across the subcortical system, age shows broadly negative cross-sectional associations with regional volume after adjustment for sex, ICV, and SNR, supporting the prediction of regionally selective negative subcortical age associations (Table 4 and Supplementary Table S1). FDR-significant negative linear age associations are observed for the hippocampus, amygdala, caudate, putamen, nucleus accumbens, total subcortical GM, and cerebellum. This overall pattern is consistent with prior adult lifespan MRI studies showing preferential age sensitivity in limbic and striatal structures rather than uniform subcortical decline.

**H1a-i.**  *Prediction: Significant negative linear age associations are expected across major subcortical structures.*

Among the significant regions, the strongest age sensitivity is observed in the striatal–limbic cluster [47]. The nucleus accumbens and caudate show the largest unique age contributions in the present dataset, followed by the amygdala. The putamen, cerebellum, and the subcortical GM composite show moderate effects. Hippocampal age sensitivity is smaller but remains robust. The prominence of striatal findings is compatible with prior evidence that ventral and dorsal striatal volumes are sensitive to ageing and clinically relevant cognitive decline [48,49,50]. The hippocampal cross-sectional age association corresponded to an estimated 0.23% lower volume per year in the fitted model. This magnitude is lower than many longitudinal estimates of hippocampal change, which is expected because cross-sectional analyses capture between-person differences rather than within-person atrophy associations. The present data therefore suggest that the hippocampus is age sensitive but not uniquely dominant; several striatal structures show larger age-related effect sizes than the hippocampus. The thalamus shows an FDR-significant negative association in absolute volume, but this result is excluded from the primary inferences because of high segmentation variance (CV > 40%). Although age-related thalamic decline has been reported previously, the present absolute volume estimate is best treated as provisional, given the segmentation instability in this dataset (note, however, that the thalamic asymmetry index remains highly stable and is retained in the H3 analyses (Section 3.4).

Cerebellar volume also shows a significant negative cross-sectional age association. This finding accords with earlier MRI volumetric evidence that older participants exhibit smaller cerebellar volumes, although the pattern is regionally heterogeneous rather than uniform across cerebellar subdivisions [51,52,53].

These results indicate a coherent cross-sectional pattern of lower subcortical volume at older ages, with particularly strong age sensitivity in the nucleus accumbens, caudate, and amygdala. Figure 3 visualises the fitted subcortical age associations, and Table 4 summarises the principal FDR-significant predictors, with full coefficients provided in Supplementary Table S1.

#### 3.2.2. Cortical Lobar Volumes and Insular Cortex

**H1a-ii ∣ H1c.**  *H1a-ii: The parietal lobe will show a nonlinear quadratic association with a midlife peak. H1c: The insular cortex and striatal structures will show the highest age sensitivity.*

FDR-significant negative linear age associations are observed in the occipital lobe and temporal lobe. In contrast, aggregate cortical GM shows no significant association with age. Frontal lobe volume shows a significant positive linear association with age. This definition is cortical GM, not an explicit GM/WM composite; the frontal total matches the sum of its constituent gyri within measurement error. Given that frontal GM typically decreases with age, this positive cross-sectional association should be interpreted cautiously and may reflect sample composition rather than a true GM increase. The insular cortex shows the strongest cortical age sensitivity in the full model, ranking third overall across all 19 regions behind the nucleus accumbens and caudate (H1c). This pattern is consistent with evidence that the insula is especially vulnerable to adult ageing, plausibly reflecting its integrative role in salience, interoceptive, and autonomic networks, although the present data do not support mechanistic inference [5].

**Figure 4:**
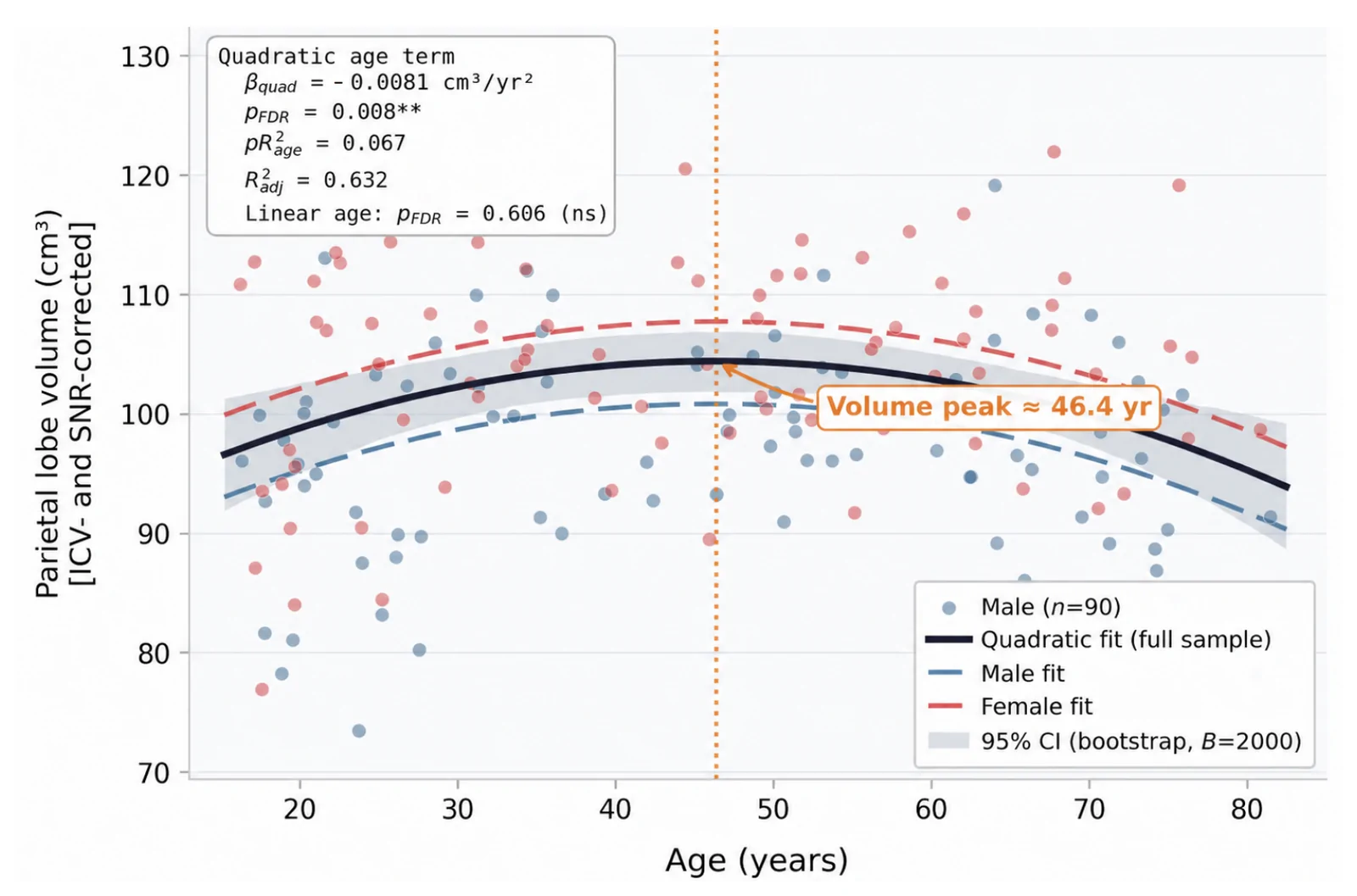
Quadratic cross-sectional age association of parietal lobe volume. The solid black curve and grey band represent the fitted association and bootstrapped 95% CI (B=2000), respectively. The fitted curve peaks at 46.4 years (95% CI: 40.6–51.3 years; vertical dotted line) and is lower at older ages beyond this point. Dashed curves denote sex-specific fits (male: blue; female: red); ** $p_{\mathrm{FDR}}<0.01$;

The parietal lobe is the only region in the 19-region analysis to show FDR-significant quadratic curvature [54]. Its linear age term is non-significant, but the quadratic term is significant, yielding an inverted-U association with a fitted vertex at 46.4 years; Figure 4, H1a-ii). This mid-fifth-decade peak is broadly compatible with evidence for the prolonged maturation of the association cortex extending into early-to-mid adulthood before later decline [55,56], but it should not be interpreted as a precise biological transition point. At the same time, the finding does not support a simple last-in, first-out account in which late-maturing cortex necessarily shows the earliest monotonic decline [57,58].

**Figure 5:**
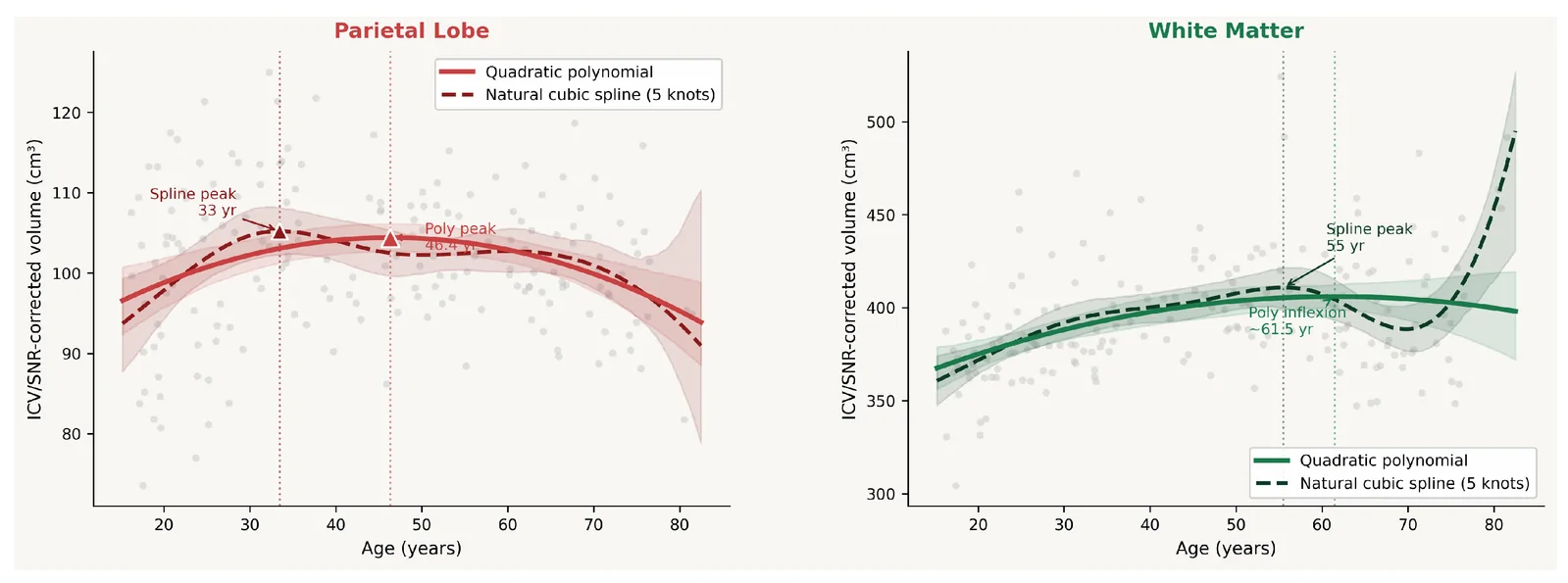
Comparison of quadratic polynomial and natural cubic spline fits for parietal lobe and WM volume. Grey points show partial residuals, and shaded bands denote bootstrapped 95% CIs (B=1000). Splines used five age-based knots (10th–90th percentiles).

**Figure 6:**
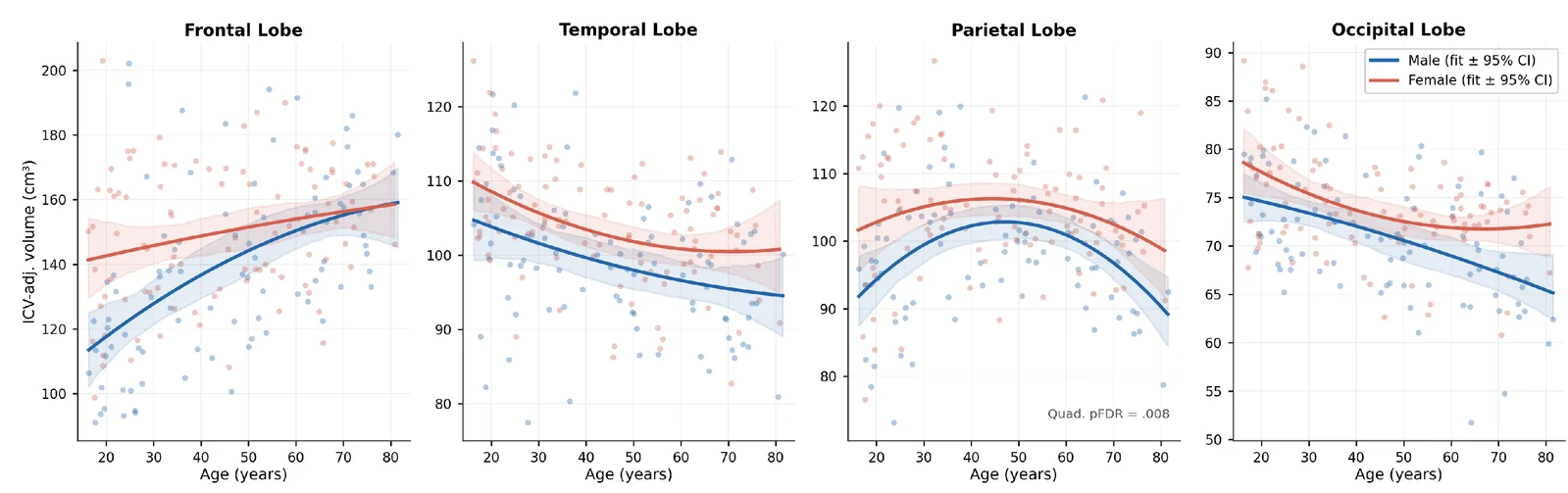
Sex-stratified cross-sectional age associations for the four lobes.

To assess sensitivity to functional form, quadratic polynomial and natural cubic spline (five knots at age deciles) fits are compared for parietal lobe age association (Figure 4 and Figure 5). Whilst both approaches agreed on the broad association shape, a ∼13-year discrepancy in parietal peak age estimates (33 vs. 46.4 years) indicates that vertex point estimates are sensitive to model specification and should not be over-interpreted. This sensitivity supports the use of the quadratic model as a parsimonious preregistered summary of nonlinearity, while also indicating that more flexible estimators may yield different inflexion estimates in future larger-sample analyses. Sex-stratified quadratic models produce modestly divergent vertex estimates across groups (male: 47 years; female: 45 years). However, the parietal lobe remains the sole cortical region with a statistically significant nonlinear age effect following FDR correction, a pattern illustrated across both modelling approaches and both sexes in Figure 5 and Figure 6.

#### 3.2.3. White Matter Volume

**H1b.**  *Prediction: WM volume will show a nonlinear inverted-U association peaking in mid-adulthood.*

**Figure 7:**
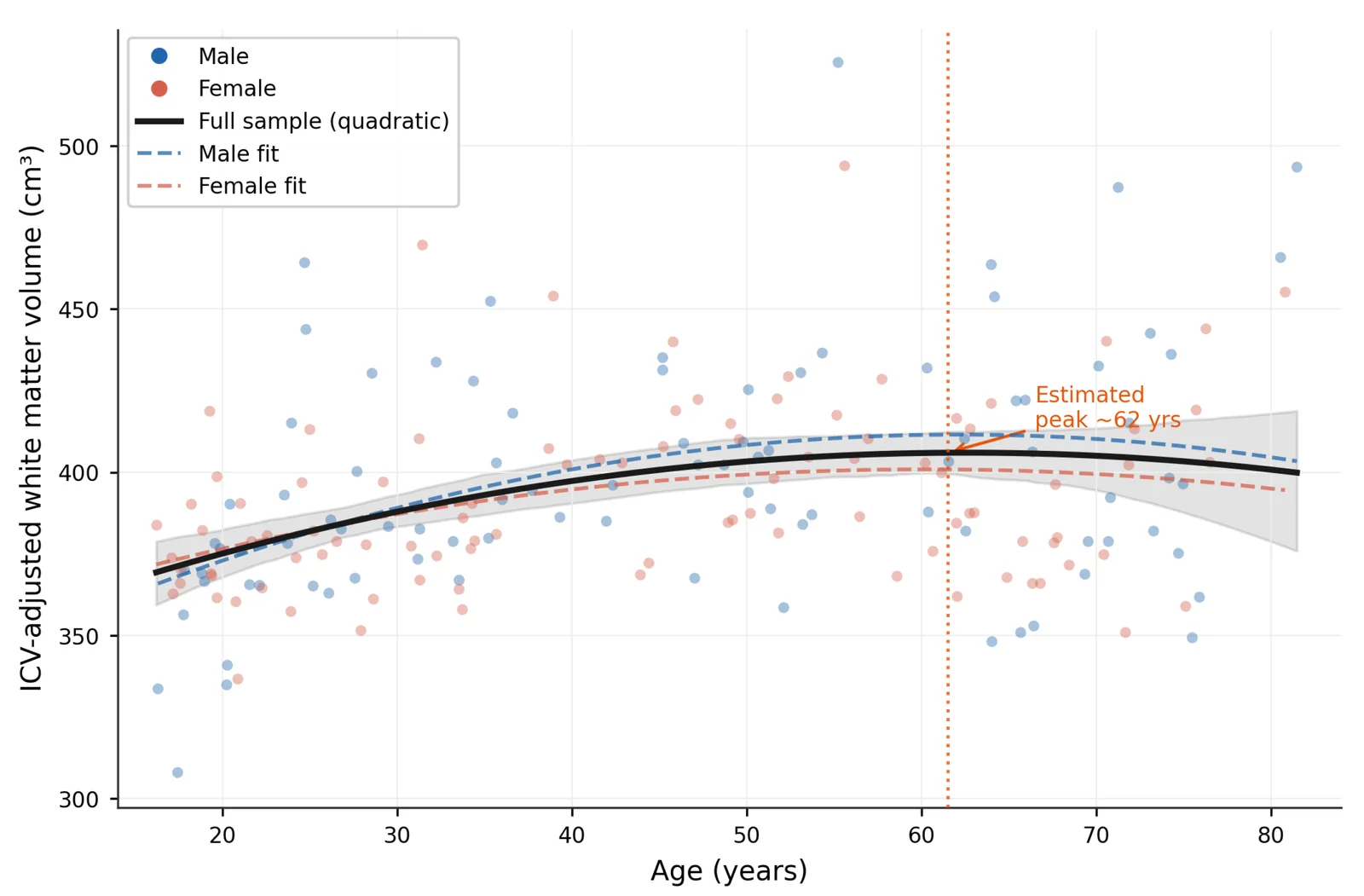
WM lifespan association fitted with a quadratic polynomial that was negative ($\beta_{\mathrm{quad}}=-0.018\;{\mathrm{cm}}^{3}/{\mathrm{yr}}^{2}$) but did not survive FDR correction ($p_{\mathrm{FDR}}=0.174$).

WM volume is positively associated with age across most of the sampled age range. Although the quadratic polynomial suggests lower fitted volume beyond a turning point at approximately 61.5 years, the quadratic term does not survive FDR correction, and this estimate should therefore be interpreted cautiously (Figure 7). Nevertheless, both the quadratic model and an independent natural cubic spline (five knots; Figure 5) identify a qualitatively similar pattern of lower fitted volume beyond this age, providing convergent but tentative cross-sectional evidence that WM volume is lower, on average, beyond the sixth decade, despite differences in the estimated turning-point age [59].

#### 3.2.4. Synthesis and Evaluation of Regional Age Associations (H1)

The H1 analyses indicate that cross-sectional age associations with regional brain volume in this sample are regionally heterogeneous rather than globally uniform. The most consistent pattern is negative cross-sectional associations between age and volume in several subcortical and lateral temporal–occipital regions, alongside a single robust nonlinear exception in the parietal lobe. Within the subcortical system, the nucleus accumbens, caudate, amygdala, putamen, hippocampus, total subcortical GM, and cerebellum show significant negative linear age associations after ICV and SNR adjustment, whereas pallidum does not. This pattern suggests that age sensitivity is concentrated in a subset of limbic–striatal structures rather than distributed evenly across all subcortical regions.

**Figure 8:**
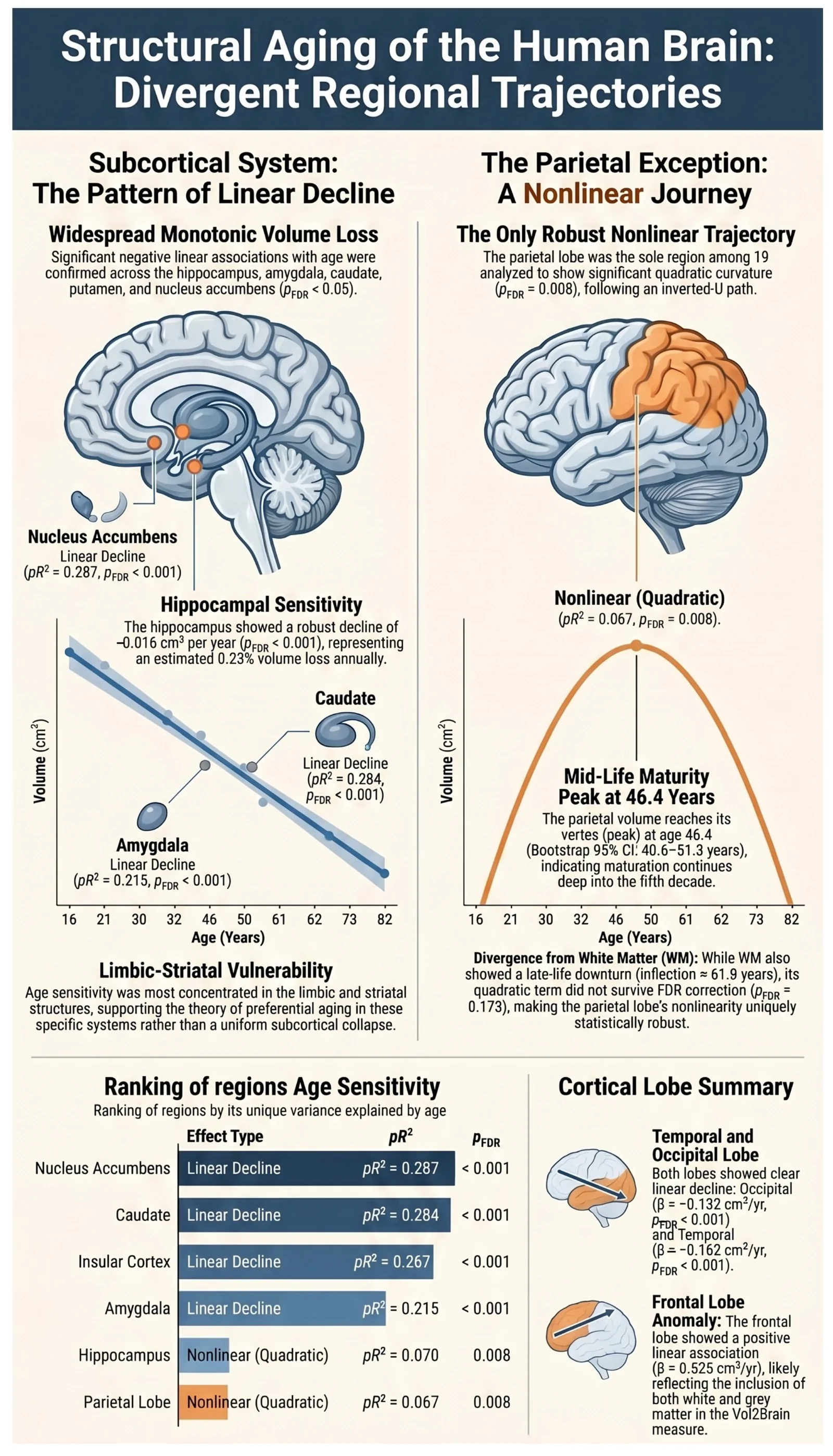
Regional brain volumes show heterogeneous cross-sectional associations with age. Key subcortical structures, including the nucleus accumbens, caudate, and amygdala, show significant negative linear age associations, whereas the parietal lobe displays a nonlinear inverted-U association with an estimated vertex at approximately 46.4 years.

**Figure 9:**
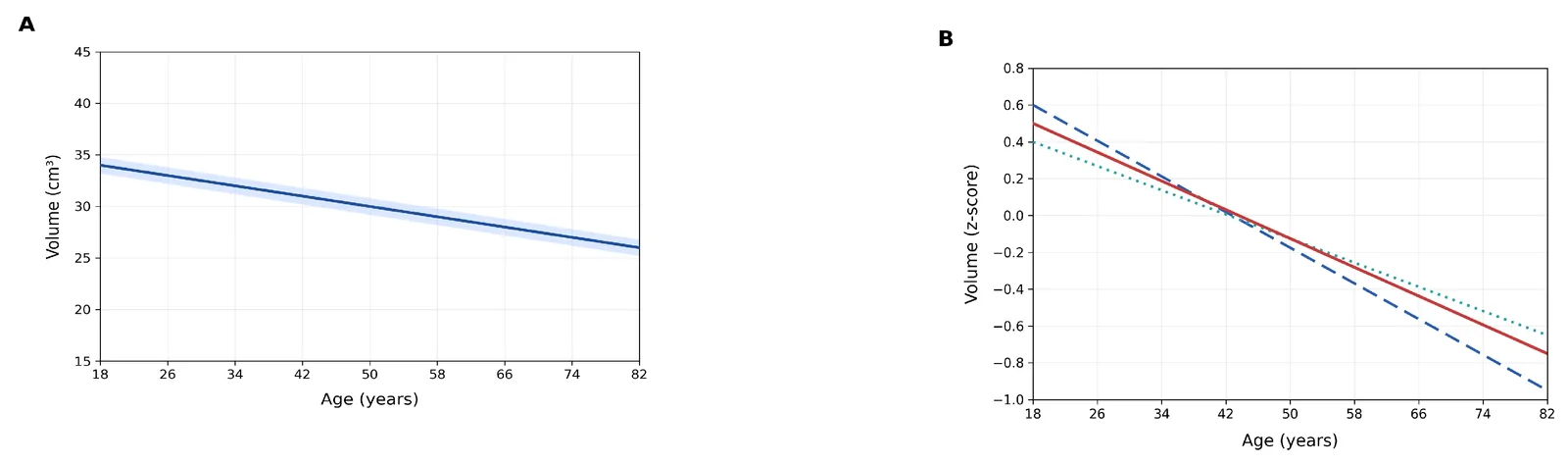
(**Panel A**): Insular volume with a linear fit (quadratic term non-significant, $p_{\mathrm{FDR}}$ = 0.774; (**Panel B**): Standardised residual age associations (z-scores) for the insular, nucleus accumbens, and caudate volumes, facilitating comparison of age-related association shapes independent of absolute volume.

The strongest age effects by partial $R^{2}$ in the full model are observed for nucleus accumbens and caudate, with the insula ranking third (Figure 8). This ordering is informative but requires careful interpretation. Partial $R^{2}$ quantifies the proportion of residual variance uniquely explained by age after covariate adjustment; it is therefore sensitive to a region’s within-sample variability, not solely to the absolute rate of volumetric change. When nucleus accumbens, caudate, and insula are plotted on a common standardised scale (Figure 9, Panel B), their cross-sectional age associations follow broadly parallel patterns, confirming that the three regions show proportionally comparable negative age associations. The higher partial $R^{2}$ values for striatal structures are more plausibly attributable to their substantially lower within-sample variability (accumbens SD = 0.2 cm^3^; caudate SD = 1.3 cm^3^) relative to the insula (SD = 5.6 cm^3^), which affords greater statistical precision in detecting age effects of similar magnitude. Accordingly, the nucleus accumbens and caudate should not be characterised as exhibiting fundamentally greater age sensitivity than the insula; rather, their volumetric homogeneity across participants renders equivalent age-related change more statistically detectable.

The parietal and WM spline comparisons speak directly to whether the quadratic parameterisation itself drives the reported nonlinear pattern. In both regions, the qualitative shape of the association (an inverted-U for the parietal lobe; a rise through most of the sampled range followed by an apparent downturn beyond the sixth decade for WM) is preserved under a substantially more flexible spline basis, whereas precise turning-point estimates are not, and should be read as approximate rather than exact. This check is restricted to the two regions in which nonlinearity is specified *a priori*; it is not extended to the remaining 17 regions, in which the quadratic term is non-significant. A non-significant quadratic coefficient indicates an absence of detectable curvature of that specific form and does not, on its own, establish linearity under every plausible functional form. We treat this as an open question, addressed further in the Section 4.

### 3.3. Sex Differences in Regional Brain Volume (H2a–H2d)

#### 3.3.1. ICV Correction and the Direction of Sex Effects (H2a–H2c)

Sex is among the most prevalent predictors of regional volume. FDR-corrected significance is observed in 12 of the 19 regions examined (Supplementary Table S1). After adjustment for age, ICV, and SNR, females exhibit significantly greater volumes in 10 grey-matter-dominant regions. These include total GM, cortical GM, hippocampus, cerebrum, pallidum, subcortical GM, and the frontal, temporal, parietal, and occipital lobes. Among these, the largest effects are observed for total GM, cortical GM, and hippocampus. The thalamus shows a significant female advantage in absolute volume but is excluded from primary H2 volume interpretation due to high absolute-volume CV (53.4%); thalamic AI is retained for H3 due to demonstrated within-subject stability (bilateral r=0.97, 0 failures, SD=11.4%).

**Figure 10:**
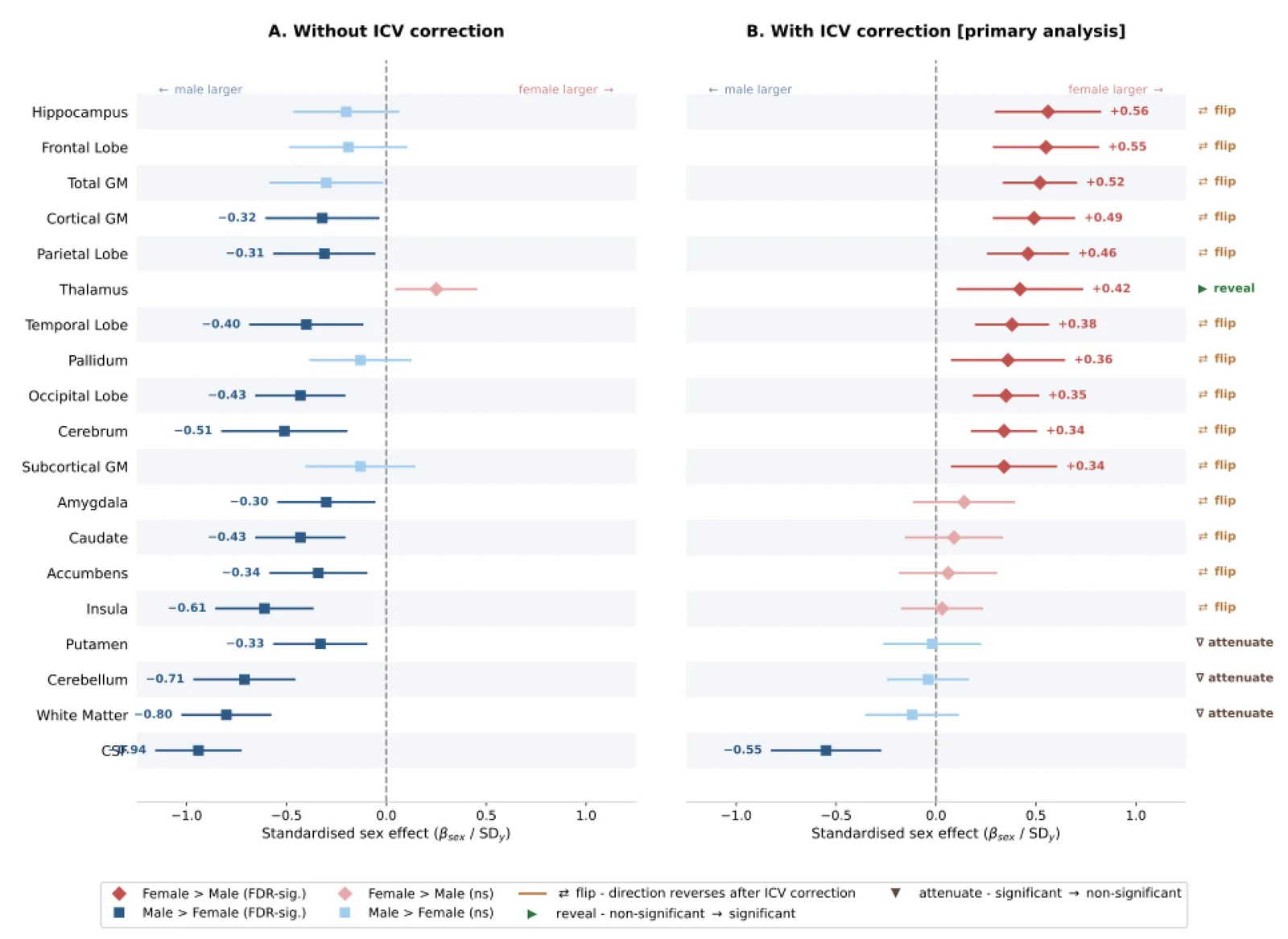
Standardised sex effects ($\beta_{\mathrm{sex}}/{\mathrm{SD}}_{Y}$; positive values: female > male). Panels (**A**,**B**): without and with ICV correction respectively. CSF is the sole region retaining male > female direction after correction.

In contrast, males show significantly greater CSF volume after ICV correction, representing one of the stronger sex effects in the dataset by partial $R^{2}$. No FDR-significant sex effects are detected for the WM, cerebellum, insula, caudate, putamen, nucleus accumbens, or amygdala. Before ICV adjustment, males show larger absolute volumes across the majority of regions (Table 3), consistent with the observed 10.4% male ICV advantage in this sample. Following ICV correction, 14 of 19 regions reverse direction, with females showing larger brain-size-adjusted volumes. This reversal is visualised in Figure 10, which contrasts unadjusted and ICV-corrected standardised sex effects across all 19 structures.

These findings support H2a and H2b: after regression-based ICV adjustment, females show larger adjusted volumes in several grey-matter-dominant regions, whereas males show larger CSF volume. H2c is also supported, as no FDR-significant sex effects remain in WM or cerebellum after ICV correction [11].

#### 3.3.2. Sex × Age Interactions (H2d)

**Figure 11:**
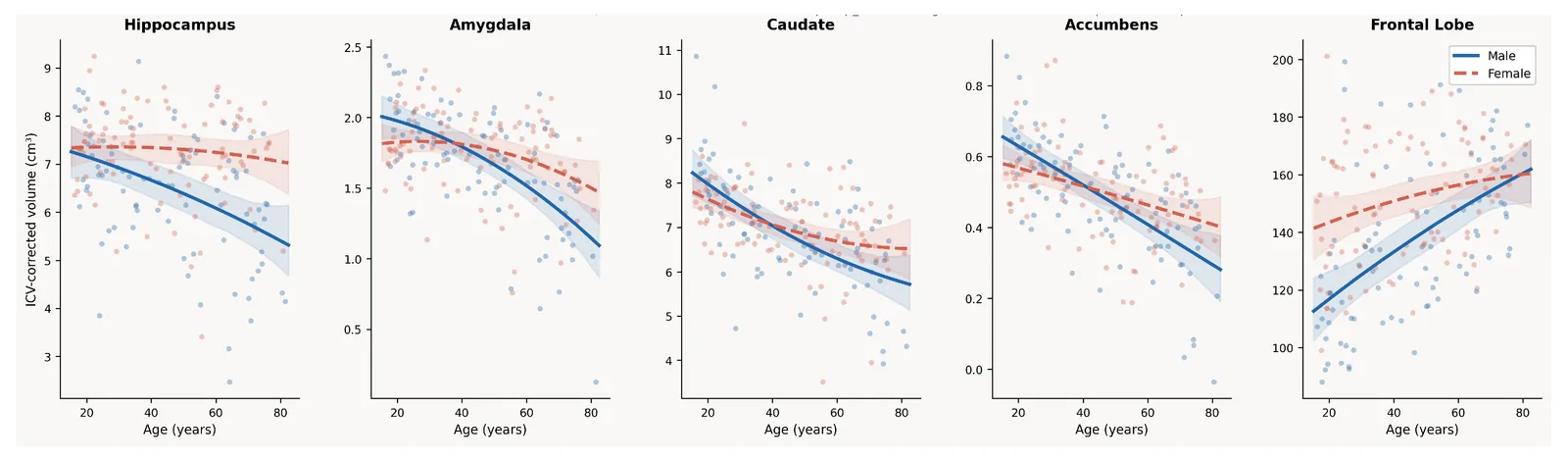
Sex × age interaction patterns for five FDR-significant regions (male: solid blue; female: dashed red; $\beta_{\mathrm{int}}$: sex × age interaction coefficient.

Sex × age interaction terms are evaluated across all 19 regions, with 5 regions demonstrating FDR-significant effects. These include four subcortical structures, namely the hippocampus, amygdala, caudate, and nucleus accumbens, as well as one cortical region, the frontal lobe. As illustrated in Figure 11, interaction patterns are visualised using ICV- and SNR-adjusted partial residuals. In the subcortical regions, positive interaction coefficients indicate shallower negative cross-sectional age associations in females compared with males, directly supporting the directional prediction of H2d. This pattern suggests a relative attenuation of the negative age association in volume in females within key subcortical systems [60,61].

**Figure 12:**
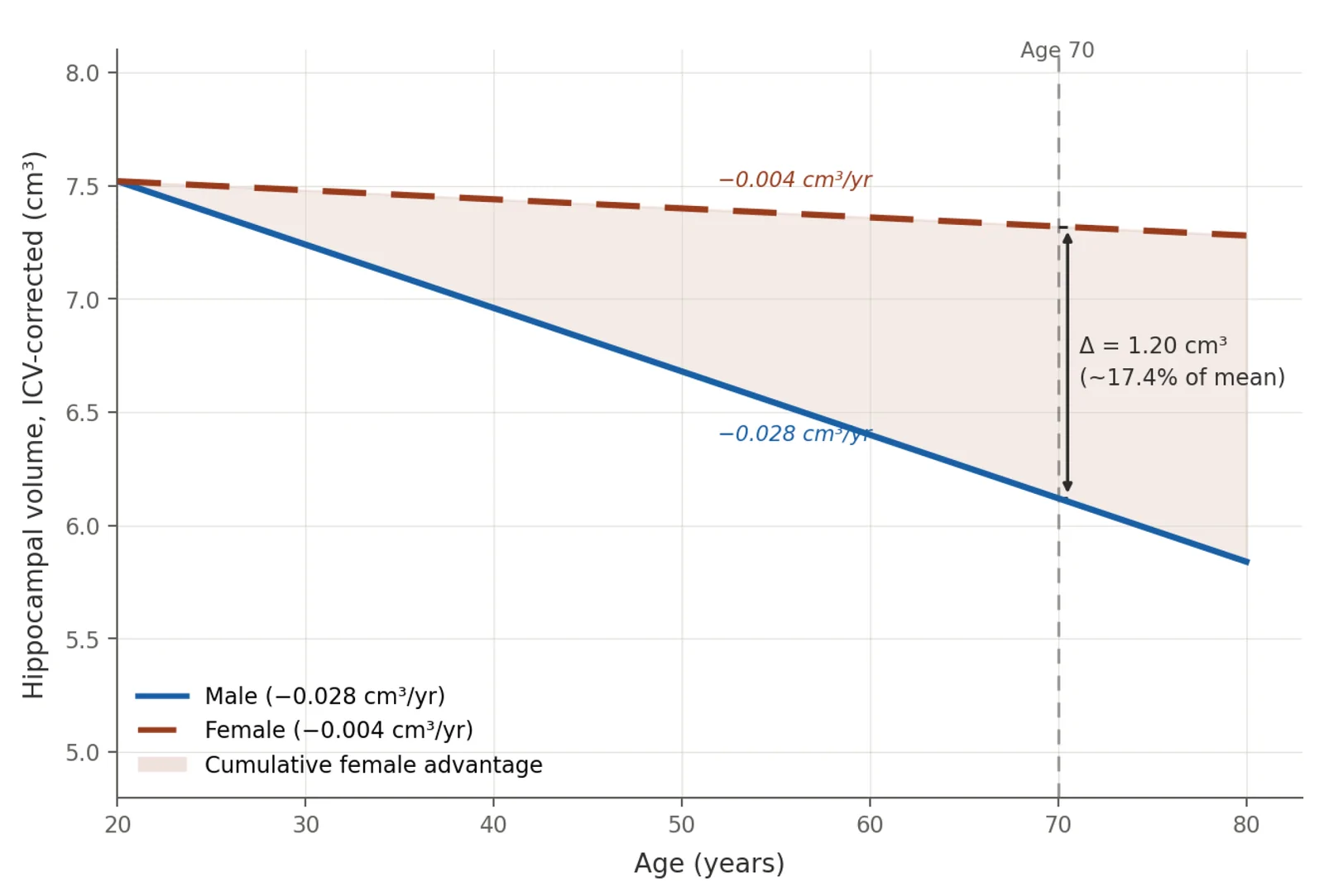
Modelled sex-by-age interaction in ICV-corrected hippocampal volume. Males (solid blue) showed a steeper negative cross-sectional age association than females (dashed red), yielding a significant interaction effect. The shaded region denotes the cumulative divergence in fitted volumes, reaching 1.20 cm^3^ by age 70.

The hippocampus provides the clearest illustrative example of a significant sex-by-age interaction in this dataset (Figure 12) [62]. The modelled cross-sectional association, anchored at a common intercept at age 20, yields a steeper age-related slope in males and a substantially shallower slope in females, with a significant interaction term. Although statistically significant, the interaction accounts for approximately 4.4% of residual variance and should be regarded as a modest effect. The shaded region in Figure 12 visualises the cumulative divergence implied by these fitted cross-sectional slopes, projecting a between-person gap of 1.20 cm^3^ (∼17.4% of the sample mean) by age 70. This pattern is consistent with evidence that hippocampal age associations may differ by sex; however, two interpretive constraints may be noted. First, this is a cross-sectional interaction and reflects between-person age differences rather than within-person longitudinal associations. Second, the common intercept at age 20 is a modelling assumption; if the youngest age stratum is unevenly represented by sex, the anchor point and projected divergence may not be fully reliable.

Comparable female-shallow cross-sectional age associations are observed in the amygdala, caudate, and nucleus accumbens. By contrast, the frontal lobe showed a negative interaction coefficient, indicating a steeper positive age association in males than in females, rather than a female advantage in grey-matter preservation. The remaining 14 regions show no FDR-significant sex × age interaction. These findings support H2d in a qualified, region-specific manner. Females exhibit shallower fitted cross-sectional age slopes in four of the nine subcortical regions examined.

The fifth significant interaction is observed in the frontal lobe. As the atlas-defined frontal regional-volume outcome contains no explicitly exported frontal-WM measure, this result cannot be attributed to the arithmetic inclusion of frontal WM. It most probably indicates a sex difference in the fitted cross-sectional frontal regional-volume association and is examined in Section 3.3.4.

In this context, the H2d findings complement H2a by indicating that sex differences in this sample reflect not only baseline ICV-adjusted volumetric differences, but also sex-specific cross-sectional age associations in selected subcortical structures.

#### 3.3.3. Synthesis of Sex Effects (H2 Verdict)

Sex is the strongest predictor overall, with FDR-significant main effects in 12 of 19 regions and interactions in 5 regions after controlling for age, ICV, and SNR (Figure 2 and Figure 10; Table 5 and Supplementary Table S1). After ICV adjustment, females show larger volumes in 10 grey-matter-dominant regions, including total GM, cortical GM, hippocampus, pallidum, subcortical GM, and the frontal, parietal, temporal, and occipital lobes, whereas males showed larger CSF volume. No significant sex effects remain in WM or cerebellum after correction, supporting H2c.

Sex × age interactions reveal shallower fitted cross-sectional age slopes in females within four subcortical regions (reflecting between-person differences), namely the hippocampus, amygdala, caudate, and nucleus accumbens [63]. By contrast, the frontal lobe shows a steeper positive age association in males than in females (male slope +0.749 cm^3^/year vs. female +0.301 cm^3^/year; interaction β=−0.448, $p_{\mathrm{FDR}}$ = 0.034). H2d is pre-specified to test whether females show shallower negative age slopes than males, a pattern consistent with relatively preserved female grey-matter volume with age. The frontal interaction does not instantiate this pattern: both sexes show positive slopes, and the interaction reflects a steeper increase in males rather than a shallower decrease in females. We therefore do not count this interaction as evidence for or against H2d, whose directional logic is formulated for regions undergoing net volumetric decline; we instead treat it as a separate, sex-specific finding in frontal grey matter, discussed on its own terms in Section 3.3.4.These interaction effects compound baseline female volume advantages, giving rise to a larger fitted sex difference at older ages (Figure 11).

**Figure 13:**
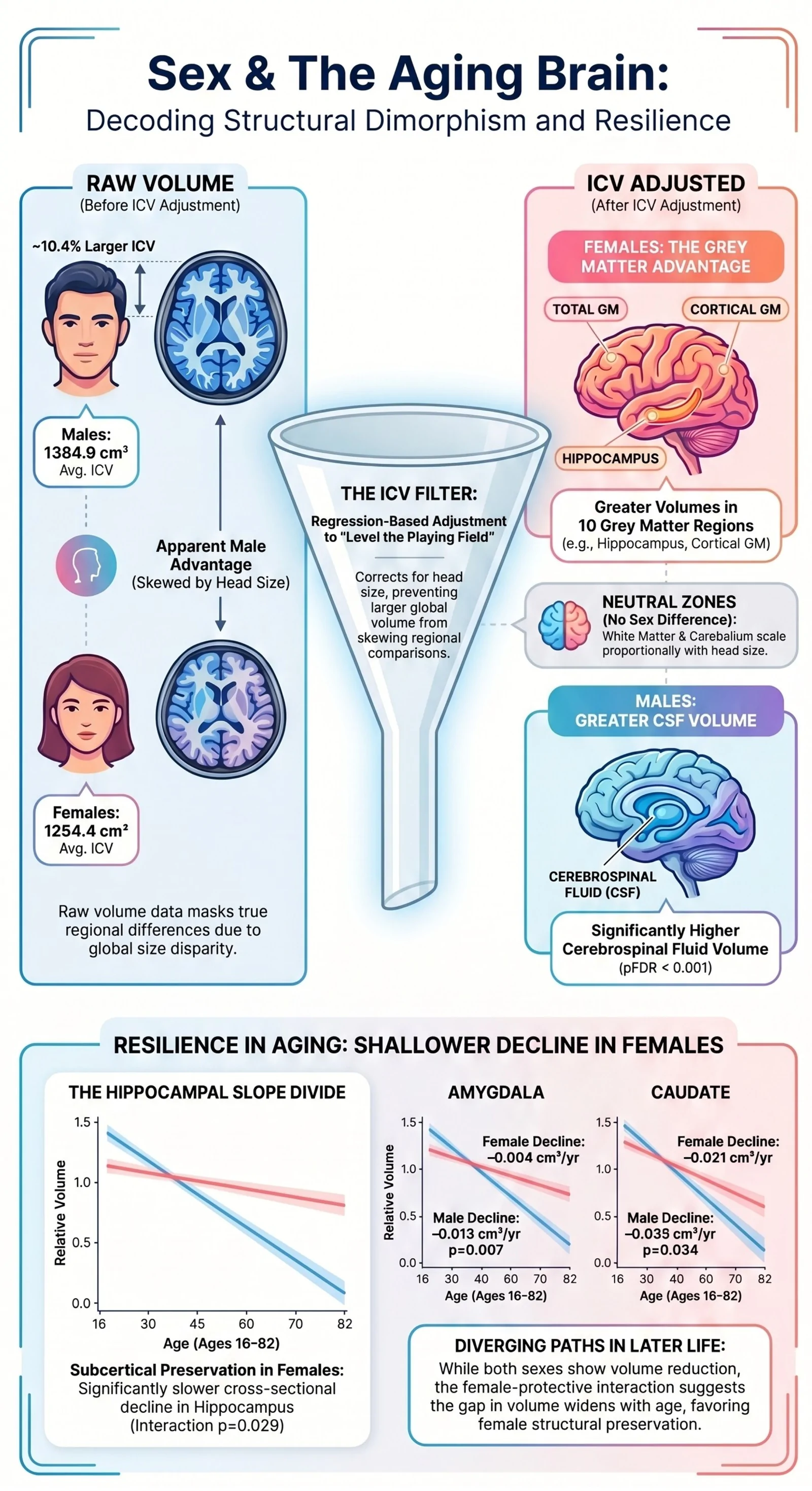
Sex-dimorphic variation and cross-sectional age associations across the adult brain. Females exhibit greater ICV-adjusted grey-matter volumes, whereas males show greater CSF volume. Significant sex-by-age interactions indicate a shallower negative cross-sectional age association in female hippocampal, amygdalar, caudate, and nucleus accumbens volumes.

H2a (female GM advantage) and H2b (male CSF advantage) are supported. H2c (no cerebellar or WM effects) is also supported, whereas H2d (female-favouring sex × age interactions) is only partially supported, specifically within subcortical regions. These ICV-adjusted patterns demonstrate sexually dimorphic cross-sectional volume-age associations primarily within subcortical structures, although limited statistical power for interaction terms warrants independent replication (Figure 13) [64].

#### 3.3.4. The Frontal Positive Age Association

The frontal lobe shows both a significant positive linear age association and a significant sex × age interaction (Table 5). Section 2.3 verifies arithmetically that this ROI is a component of cortical GM, not a GM/WM composite: the frontal total matches the sum of its 17 constituent gyri to within 0.03 cm^3^ per participant, and the frontal, temporal, parietal, occipital, insular, and limbic lobes together reproduce the pipeline’s independently computed cortical GM volume to within 0.02 cm^3^ per participant. (mean absolute difference 0.005 cm^3^). This rules out the possibility that the reported frontal figure is a straightforward arithmetic composite of separately tabulated grey- and white-matter sub-totals. It does not, on its own, rule out misclassification occurring further upstream, within the pipeline’s voxel-level tissue-classification step.

Aggregate cortical GM shows no significant age association, whereas the temporal and occipital lobes show significant negative linear cross-sectional associations. The frontal association should therefore be interpreted as a pipeline- and atlas-defined regional-volume finding, rather than as evidence that frontal GM increases with age or as a confirmed regional exception to established cortical ageing patterns [2].

We treat this as a provisional finding rather than a confirmed regional exception, for three reasons. First, the effect is carried predominantly by males (male slope +0.749 cm^3^/year vs. female +0.301 cm^3^/year; interaction β=−0.448, $p_{\mathrm{FDR}}$ = 0.034; Table 5) and is weak in females alone. So it may reflect a sex-specific process rather than a general property of frontal cortex. Second, the cross-sectional design confounds age with cohort, so the association may reflect generational differences rather than within-person ageing. A longitudinal follow-up can distinguish these. Third, the stark divergence from all other cortical results above requires cross-pipeline replication. A comparison against an independent pipeline, e.g., FreeSurfer, would help test this.

#### 3.3.5. Potential Neurobiological Contributors to Sex-Differentiated Regional Ageing

The present cross-sectional structural data identify sex-differentiated volume–age associations but cannot establish the biological mechanisms underlying them. The AgeRisk dataset contains no detailed vascular, inflammatory, or metabolic biomarkers [26]. The following interpretation is therefore literature-informed and hypothesis-generating rather than directly tested in the present sample.

The hippocampus, amygdala, caudate, and nucleus accumbens are key components of limbic–striatal circuits supporting memory, affective processing, reward learning, and motivated behaviour. Their differential cross-sectional associations with age in females and males may reflect the cumulative and interacting influence of endocrine, vascular, immune, and metabolic processes across the life course, rather than a single sex-specific biological process.

Gonadal-steroid exposure changes substantially across adulthood, particularly during reproductive transitions and later life, and may interact with cerebrovascular signalling and vascular remodelling. Recent work has highlighted sex variation in mechanisms of cerebrovascular ageing, including the potential relevance of menopause, sex hormones, and vasoactive signalling [16]. Such processes could plausibly influence regional brain maintenance through altered perfusion, endothelial function, blood–brain barrier integrity, and vascular reactivity. However, as the present analysis included none of these measures, the observed regional volume differences cannot be ascribed to hormonal or vascular mechanisms.

Sex-related variation in immune signalling may also interact with endocrine and vascular ageing, potentially influencing neural maintenance and susceptibility to age-related change. As no direct markers of peripheral or central inflammation are available, this explanation remains hypothesis-generating rather than a demonstrated mediator of the associations reported here.

Metabolically, sex-dependent regulation of glucose transport, glycolysis, and mitochondrial function has been proposed as a contributor to sex-differentiated brain metabolic vulnerability with ageing [17]. Individual variation in cardiometabolic health could differentially affect structural maintenance in limbic–striatal systems across adulthood due to their high energetic demands. Direct metabolic measures, including glucose, insulin, lipid, body-composition, mitochondrial, and regional metabolic-imaging measures, are unavailable; consequently, this hypothesis cannot be evaluated.

The dataset records self-reported gender as female or male. This variable does not measure chromosomal sex, circulating hormone concentrations, reproductive stage, gender identity beyond the available categories, or sex-related social and environmental exposures. The results should therefore be interpreted as differences associated with the available female/male classification, rather than as evidence that any single biological mechanism determines regional brain ageing.

### 3.4. Hemispheric Asymmetry

#### 3.4.1. H3a: Population-Level Directional Lateralisation

**H3a.**  *Prediction: Established structural lateralisation patterns are confirmed at the population level across bilateral regions.*

Seven of the original 19 regions are excluded from the confirmatory family because of methodological and epistemic constraints. Four global tissue classes, namely total GM, cortical GM, subcortical GM, and cerebrospinal fluid, are omitted because the Vol2Brain pipeline does not provide hemisphere-specific segmentations, precluding valid AI computation. Total WM, the cerebellum, and the occipital lobe are excluded to maintain statistical coherence within the confirmatory testing framework.

Thalamic absolute volume is excluded from H1/H2 due to high between-subject CV (53.4%) but thalamic AI is retained for H3 because AI is a within-subject ratio that cancels correlated bilateral scaling error (bilateral r=0.97, 0/187 failures, 0/187|AI|>100%, AI SD=11.4%). CV is not interpretable for AI when mean approaches zero, so stability is evaluated via SD and failure rate rather than CV.

**Figure 14:**
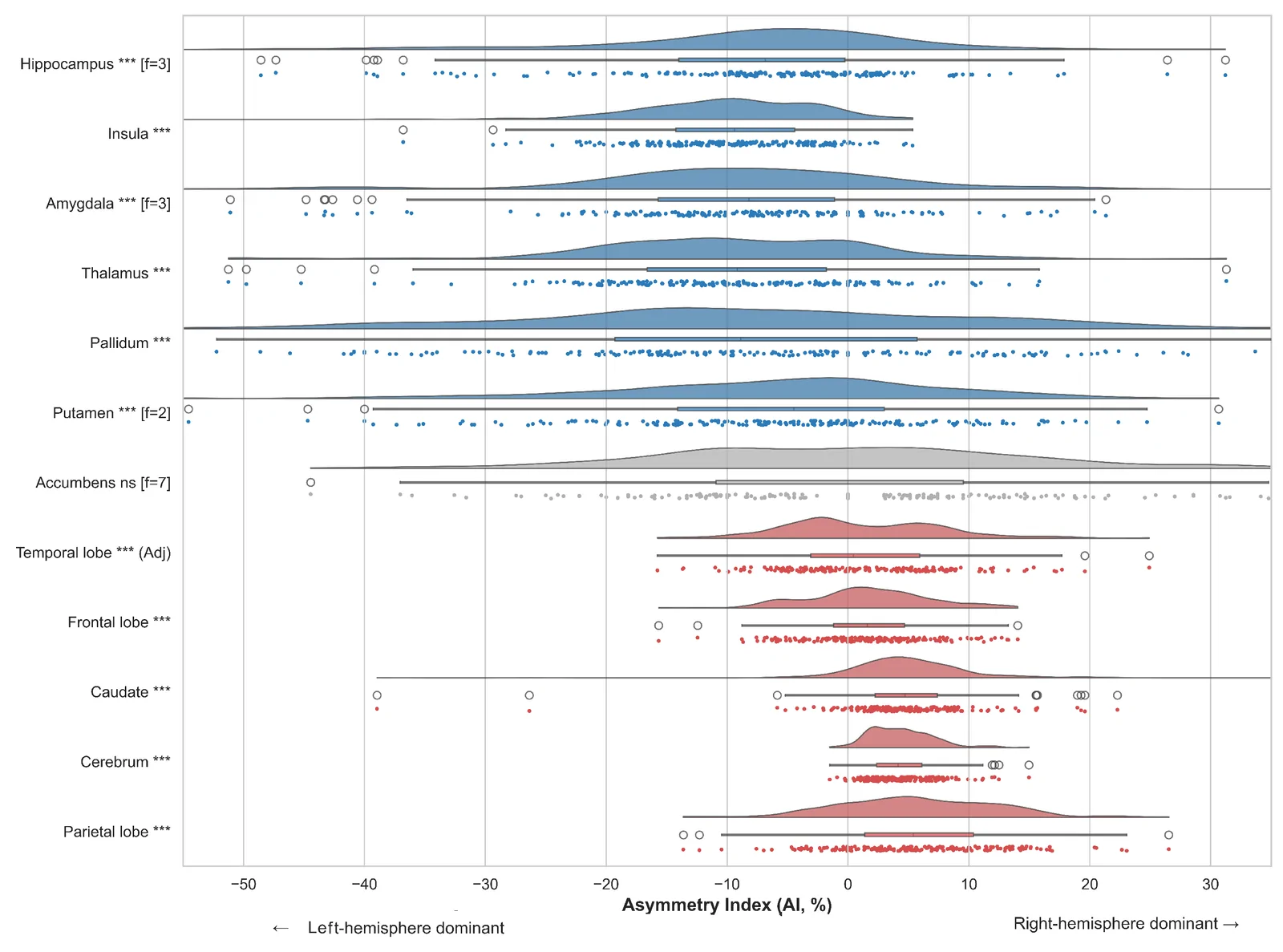
Population-level hemispheric asymmetry indices across 12 bilateral structures. Raincloud plots display AI, distributions with raw observations and boxplots (blue: significant leftward asymmetry; red: significant rightward asymmetry; grey: non-significant asymmetry.

Population-level hemispheric asymmetry is examined across 12 pre-specified bilateral structures using continuous AI, with inference based primarily on one-sample tests against zero under Bonferroni correction (Table 6; Figure 14). Significant directional lateralisation is observed in 10 of the 12 confirmatory structures in the unadjusted analyses, indicating that hemispheric volume asymmetry is a common feature of the present sample rather than an isolated property of a few regions. These effects are large in magnitude for several subcortical and cortical regions and remain directionally stable in covariate-adjusted sensitivity analyses, supporting H3a.

Leftward asymmetry (AI<0) is observed in the hippocampus, amygdala, insula, thalamus, pallidum, and putamen, all Bonferroni-significant in the primary analysis. Rightward asymmetry (AI>0) is observed in the frontal lobe, cerebrum, caudate, and parietal lobe, all of which survive correction. These findings are consistent with established large-scale neuroanatomical asymmetry patterns and indicate that the AI formulation recovers biologically plausible directional effects across both cortical and subcortical systems.

Two regions do not meet the confirmatory significance threshold in the unadjusted tests. The nucleus accumbens shows no evidence of lateralisation, with CIs spanning zero and no meaningful directional trend. The temporal lobe likewise fails to survive the unadjusted confirmatory threshold, despite a modest positive mean AI, indicating that any directional asymmetry in this region isweaker and more age-sensitive than in the other confirmatory structures [65].

Distributional diagnostics justify the use of mixed inferential procedures. Shapiro–Wilk tests indicate non-normal AI distributions in 10 of the 12 structures, with only frontal and parietal AIs approximating normality. Wilcoxon signed-rank tests are therefore applied to most regions, whereas one-sample *t*-tests are used for the frontal and parietal lobes.

Segmentation failure rates are low but non-zero, occurring most frequently in the nucleus accumbens (7/187, 3.7%) and less often in the hippocampus and amygdala (3/187 each, 1.6%) and the putamen (2/187, 1.1%); all other confirmatory regions have zero failures. These low failure rates suggest acceptable robustness of the asymmetry-estimation pipeline, although results for the nucleus accumbens should be interpreted with greater caution because of elevated missingness.

The 10 structures that show significant unadjusted asymmetry retain the same directional pattern in covariate-adjusted ordinary least squares sensitivity models controlling for age, quadratic age, sex, and ICV, with all adjusted effects remaining highly significant. This indicates that the observed lateralisation patterns are not artefacts of head size, sex composition, or age-related scaling but instead reflect stable structural organisation at the population level.

The temporal lobe is the only region in which adjustment materially changed inference, becoming significant after covariate control. This suggests that age-related drift partially masked its average asymmetry in the raw, unadjusted analysis.

These results support H3a by confirming directional hemispheric lateralisation across most pre-specified bilateral structures, with strong consistency between unadjusted and adjusted analyses. There is no evidence that the principal asymmetry patterns are driven by demographic or allometric confounding, indicating robust population-level lateralisation independent of age, sex, and ICV. Table 6 summarises mean AI values, CIs, and inferential results for all 12 confirmatory structures, and Figure 14 visualises the full distribution of asymmetry indices across regions.

#### 3.4.2. H3b: Age-Related Asymmetry Reduction (Cortical Dedifferentiation)

**H3b.**  *Prediction: Posterior cortical asymmetry decreases with age (structural HAROLD), whereas subcortical asymmetry remains stable across adulthood.*

**Figure 15:**
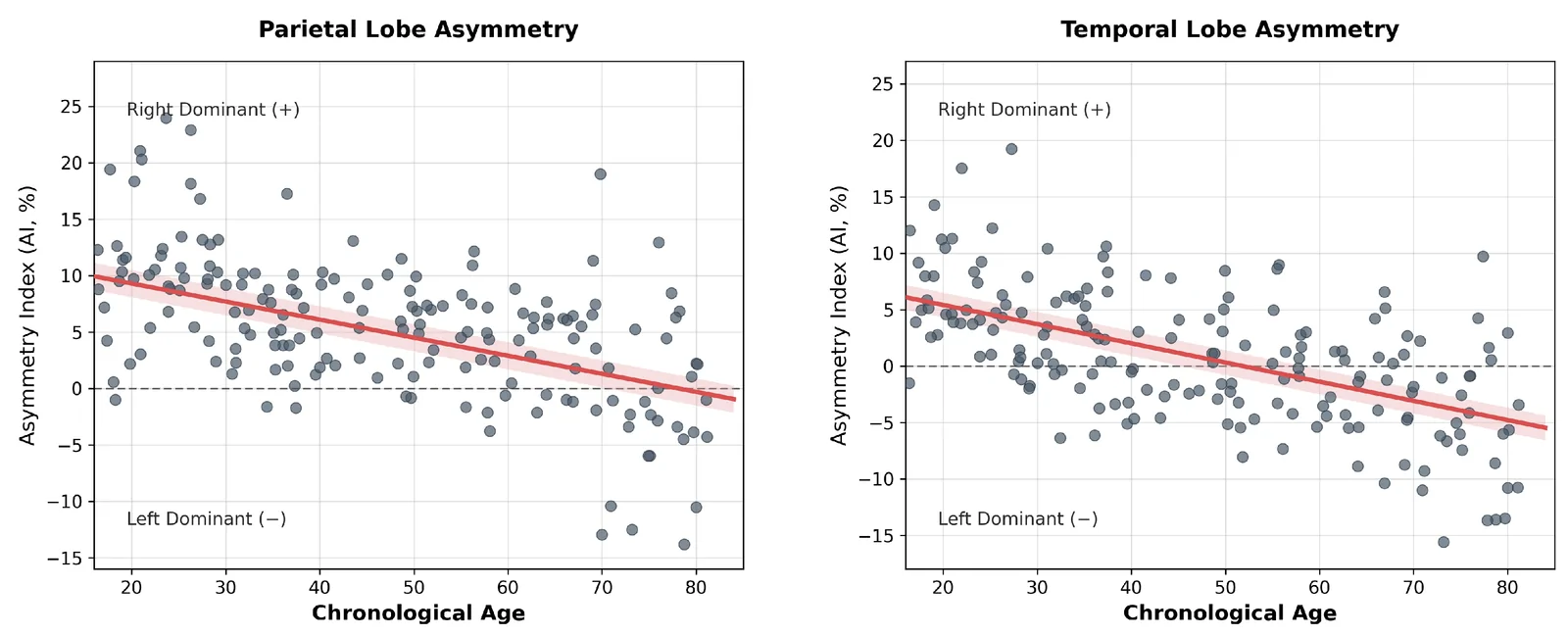
Negative cross-sectional age association with hemispheric AI for the parietal (**left**) and temporal (**right**) lobes. Scatter plots show individual AI values (%) across age; positive values indicate rightward asymmetry and the dashed line denotes symmetry (AI = 0). Red lines show OLS fits with 95% CIs. Both regions show a significant negative association between age and AI (p< 0.001).

**Figure 16:**
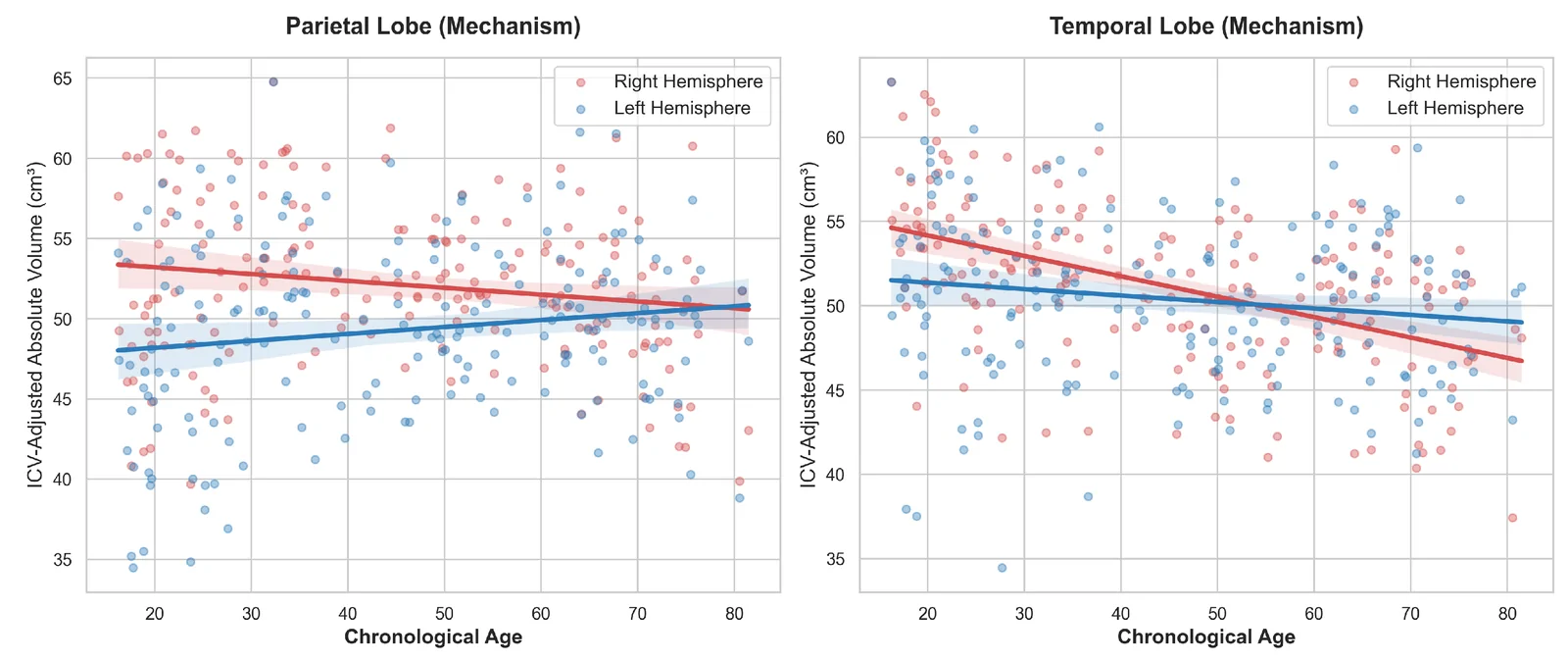
Exploratory hemisphere-specific ICV-adjusted volume associations for the parietal (**left**) and temporal (**right**) lobes. Fitted age associations indicate a steeper negative right-hemisphere age association and greater hemispheric convergence at older ages in both regions. These descriptive cross-sectional plots illustrate the volumetric patterns underlying the negative age associations with AI shown in Figure 15.

To test whether hemispheric asymmetry changes systematically with age, partial correlations are computed between age and AI across the 12 pre-specified bilateral structures, controlling for sex and ICV, with Bonferroni correction applied across regions; see Table 7 and Figure 15 and Figure 16). Five regions show significant age-related AI reduction after correction, namely the parietal lobe, temporal lobe, cerebrum, insula, and amygdala.

The strongest effects are observed in posterior cortical regions. Parietal AI shows a pronounced negative association with age, indicating a lower degree of its typical rightward asymmetry at older ages. A similarly negative association is present in the temporal lobe, consistent with age-related reduction in rightward dominance. The cerebrum also shows a significant but smaller negative association, indicating that global hemispheric asymmetry is less marked at older ages. The insula demonstrates a more modest but still Bonferroni-significant reduction in asymmetry.

Most subcortical regions do not show a significant age-related change in AI. No Bonferroni-significant age associations are observed for the thalamus^a^, pallidum, putamen, nucleus accumbens, caudate, or hippocampus, indicating relative stability of their population-level lateralisation across the sampled age range. The amygdala is the only subcortical exception, showing a significant negative age–AI association. The thalamic null result here is reported on the same basis as the significant thalamic H3a finding above (Table 6, note a): the justification for retaining thalamic AI rests on the properties of the measure itself, applied identically regardless of significance.

The amygdala is the only subcortical exception, showing a significant negative age–AI association. This weakens a strict claim of universal subcortical stability but does not alter the broader cortical–subcortical dissociation in the pattern of age-related asymmetry change. The regional distribution of these effects is therefore selective rather than global. Stronger age-related AI reduction is concentrated in posterior and association cortical regions, whereas most subcortical asymmetries are comparatively stable.

This pattern is compatible with a structural analogue of age-related hemispheric dedifferentiation, but the interpretation should remain cautious [66]. Convergence of asymmetry indices in volumetric measures does not equate to reduced functional lateralisation and should not be taken as direct validation of the HAROLD framework [67].

A subsidiary methodological check tests whether these age–AI associations are adequately characterised as linear. For the two strongest cortical findings, adding a quadratic age term does not improve model fit for parietal or temporal AI, supporting a predominantly linear age-related reduction in asymmetry rather than a threshold or midlife-transition effect.

Post hoc hemisphere-specific analyses suggest that the mechanisms underlying AI reduction are regionally heterogeneous. In the temporal lobe, asymmetry reduction is driven primarily by stronger age association in the right hemisphere than in the left hemisphere, consistent with asymmetric right-sided volume loss. Parietal convergence reflects non-parallel hemispheric patterns, with right parietal volume showing a negative association with age and left parietal volume showing a weak positive association. These exploratory analyses indicate that similar reductions in AI may arise from distinct hemispheric processes rather than a single shared mechanism [68].

Age-related AI reduction is significant under Bonferroni correction in 5 of 12 confirmatory regions: parietal lobe, temporal lobe, cerebrum, insula, and amygdala (Table 7). The negative AI–age association followed a linear pattern in all significant regions except the amygdala, which shows a significant quadratic age component, suggesting a more complex lateralisation pattern for this structure. The caudate is the sole region showing a positive age–AI association, though this does not survive Bonferroni correction. The two strongest cortical effects are illustrated in Figure 15 and Figure 16, where exploratory hemisphere-specific age associations indicate that the negative AI–age association in the parietal and temporal lobes is driven primarily by a steeper negative age association in right-hemisphere volume relative to the left. H3b is therefore partially supported: robust age-related asymmetry reduction is confirmed in posterior and association cortical regions, whereas most subcortical structures remain stable; the amygdala represents a notable subcortical exception and warrants replication before being assigned a broader theoretical role.

#### 3.4.3. Sex Differences in Hemispheric Asymmetry (H3c)

**Figure 17:**
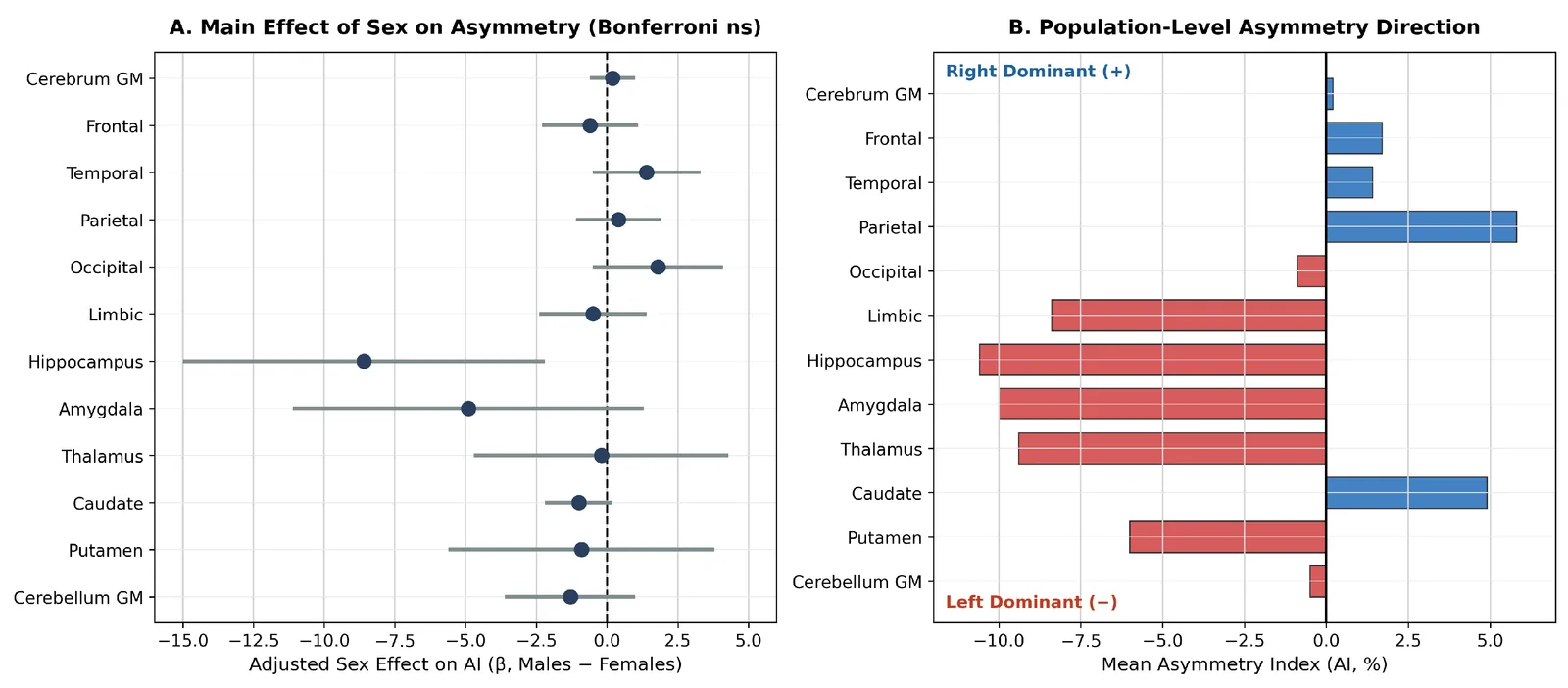
Sex effects on hemispheric asymmetry and population-level asymmetry across 12 bilateral regions. Panel (**A**): Adjusted sex effects (β, males–females) on the AI; error bars denote 95% CIs. No region survives Bonferroni correction (α=0.004). Panel (**B**): Mean AI values by region, with blue indicating rightward asymmetry and red indicating leftward asymmetry.

**H3c.**  *Prediction: To examine whether hemispheric asymmetry differed by sex, the AI is modelled across 12 pre-specified bilateral regions, including thalamus retained for AI only (absolute-volume CV =53.4% excluded for volume, AI stable r=0.97, *0* failures), using covariate-adjusted ordinary least squares, with sex as focal predictor and linear age, quadratic age, and ICV as covariates (Table 8; Figure 17).*

Bonferroni correction is applied across the 12 confirmatory tests. Under this criterion, no region yields a significant main effect of sex on AI magnitude. This finding should be interpreted as an absence of detectable sex differences under a conservative threshold, rather than as positive evidence of equivalent lateralisation between sexes; the notably wide CIs for hippocampus and amygdala indicate particularly low precision for those regions, and meaningful sex differences cannot be excluded on current evidence.

The largest uncorrected sex effect is observed in the hippocampus, raw, indicating greater rightward, or less leftward, hippocampal asymmetry in males relative to females. However, this effect does not survive Bonferroni correction and therefore cannot be interpreted as confirmatory evidence of sexual dimorphism in hippocampal asymmetry.

All remaining regions show weak and non-significant sex coefficients at the uncorrected level, including the cerebrum, frontal lobe, temporal lobe, parietal lobe, thalamus (retained for AI; bilateral r=0.97, 0 failures, SD=11.4%), caudate, putamen, and amygdala.

The absence of significant sex effects on AI contrast with the robust ICV-adjusted sex differences in absolute regional volume reported in Section 3.3. Females show larger adjusted volumes in multiple grey-matter-dominant regions, whereas males show larger CSF volume. These volumetric differences are not accompanied by systematic differences in asymmetry magnitude. This dissociation indicates that sex-related variation in regional volume endowment does not necessarily extend to the bilateral architectural organisation of those regions.

The population-level asymmetry directions established under H3a are fully consistent across sexes. Regions showing leftward asymmetry at the sample level, such as the hippocampus, amygdala, and insula, retain the same directional pattern in both males and females. Regions showing rightward asymmetry, such as the parietal lobe, caudate, and cerebrum, likewise exhibit no sex-specific reversal in lateralisation direction. The present data therefore provide no evidence that males and females differ in the basic directional architecture of hemispheric volumetric asymmetry.

An exploratory extension tests Sex × Age interaction terms for AI to assess whether the cross-sectional age association in structural asymmetry differed between males and females. No interaction survives FDR correction in any region, indicating that there is no detectable evidence for sexually dimorphic asymmetry–age associations in this sample. These interaction analyses should be regarded as underpowered and hypothesis-generating rather than definitive.

These results support H3c by showing that, after accounting for age and ICV, hemispheric asymmetry is not meaningfully moderated by sex across the confirmatory bilateral structures. Table 8 summarises adjusted sex coefficients for AI across regions, and (Figure 17) presents the corresponding forest plot of sex effects with 95% CIs.

#### 3.4.4. Synthesis of Hemispheric Asymmetry Findings (H3 Verdict)

Hemispheric asymmetry analyses demonstrate robust population-level directional lateralisation across nearly all 12 pre-specified bilateral structures, except for the nucleus accumbens; the temporal lobe reaches significance only in the adjusted model, thereby supporting H3a (Table 6). Leftward asymmetry characterises the hippocampus, amygdala, insula, thalamus, pallidum, and putamen. Rightward asymmetry characterises the frontal lobe, cerebrum, caudate, and parietal lobe. All effects are stable after covariate adjustment for age, sex, and ICV.

**Figure 18:**
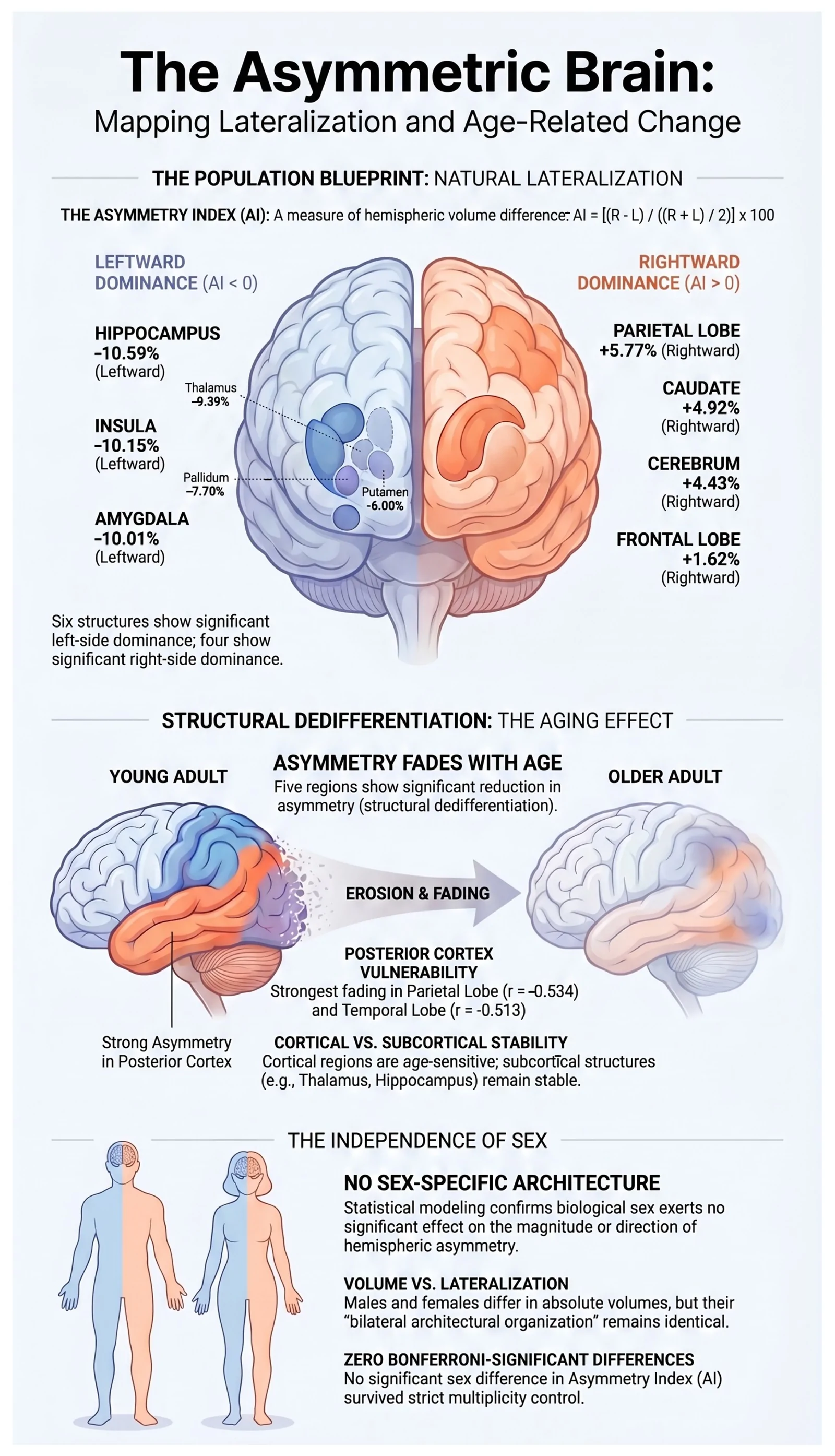
Summary of hemispheric asymmetry and age-related changes in structural lateralisation across the brain. Asymmetry patterns reveal leftward asymmetry in limbic structures and rightward asymmetry in several cortical regions. Significant age-related reductions in asymmetry are observed in the parietal and temporal lobes, whereas no significant sex effects are detected.

The negative age association in AI is selective rather than global (H3b: partially supported; Table 7). Bonferroni-significant effects are concentrated in the posterior cortex, including the parietal lobe, temporal lobe, cerebrum, and insula, with the amygdala as the sole subcortical exception. Most subcortical asymmetries show no significant cross-sectional age association (Figure 18).

Most subcortical regions do not show a significant age-related change in AI. No Bonferroni-significant age associations are observed for the thalamus, pallidum, putamen, nucleus accumbens, caudate, or hippocampus, indicating relative stability of their population-level lateralisation across the sampled age range. The amygdala is the only subcortical exception, showing a significant negative age–AI association. This weakens a strict claim of universal subcortical stability but does not alter the broader cortical–subcortical dissociation in the pattern of age-related asymmetry change.

A subsidiary methodological check tests whether these age–AI associations are adequately characterised as linear. For the two strongest cortical findings, adding a quadratic age term does not improve model fit for parietal or temporal AI, supporting a predominantly linear age-related reduction in asymmetry rather than a threshold or midlife-transition effect. The largest uncorrected sex effect is observed in the hippocampus, indicating greater rightward, or less leftward, hippocampal asymmetry in males relative to females. However, this effect does not survive Bonferroni correction and therefore cannot be interpreted as confirmatory evidence of sexual dimorphism in hippocampal asymmetry. All remaining regions show weak and non-significant sex coefficients at the uncorrected level, including the cerebrum, frontal lobe, temporal lobe, parietal lobe, thalamus, caudate, putamen, and amygdala.

No region shows significant sex differences in AI magnitude after Bonferroni correction (H3c: supported; Table 8). The largest uncorrected effect occurs in the hippocampus but did not survive multiplicity control. These findings establish reliable structural lateralisation benchmarks and selective age-related convergence primarily in posterior association cortex, with no evidence of sex-specific asymmetry architecture in this sample.

## 4. Limitations and Future Directions

This study has several important limitations that should be considered when interpreting the findings:

1. Cross-sectional design: All age-related findings reflect cross-sectional between-person differences rather than within-person longitudinal change. Phrases such as “decline”, “trajectories”, or “age-related reduction” refer exclusively to fitted cross-sectional patterns and should not be interpreted as implying individual-level biological ageing rates or causal longitudinal atrophy. Longitudinal studies are required to confirm true intra-individual change.
2. Modest sample size for interactions: With N=187, the study had limited power to detect modest sex × age interaction effects. The four significant subcortical interactions are hypothesis-consistent but provisional and require replication in larger cohorts.
3. Single-site, single-scanner cohort: All data were acquired on one 3T Siemens Prisma scanner using identical protocols, which eliminated scanner effects but limited generalizability to other field strengths, manufacturers, and acquisition parameters [69,70].
4. Age distribution limitations: Sampling was relatively sparse in the oldest age decade (n=25 for age ≥70 years), potentially reducing sensitivity to late-life effects. The convenience sample may also not be representative of the population [71,72].
5. Segmentation variability: Vol2Brain provides useful subcortical estimates but shows higher variance in thalamic volumes (CV>40%) and occasional hemisphere-specific failures (1% to 4% in the hippocampus, amygdala, and nucleus accumbens). These cases were handled transparently as missing data. A further methodological point concerns the differential treatment of the thalamus across the volumetric and asymmetry analyses: absolute thalamic volume was excluded from primary inference because of high segmentation variance, whereas the thalamic AI was retained because the underlying instability is substantially shared across hemispheres and is therefore attenuated in the derived ratio measure. This is supported by the near-collinearity of left and right thalamic volumes in this sample (r=0.973) and by the independence of thalamic AI from both absolute volume (r=0.135) and SNR (r=−0.008). We treat this as a resolved methodological distinction rather than an open limitation, though independent replication using a segmentation pipeline with established thalamic reliability (e.g., FreeSurfer’s probabilistic thalamic nuclei atlas) would further strengthen confidence in this result.
6. Absence of biomarker data: The AgeRisk imaging release does not include hormonal (e.g., estradiol and testosterone), vascular (e.g., blood pressure and arterial stiffness), inflammatory (e.g., cytokines, CRP), or metabolic (e.g., glucose and lipid profile) measures. Consequently, the mechanistic interpretation of observed sex differences in regional volume remains literature-based rather than empirically tested within this sample [73,74,75,76]. Future work integrating endocrine panels, vascular indices, and inflammatory markers with structural MRI would enable direct mediation testing of these candidate pathways.
7. Structural vs. functional asymmetry: Volumetric asymmetry indices are not equivalent to functional lateralisation measures. The present findings are compatible with, but do not validate, the HAROLD framework, which was derived from task-based fMRI.
8. Multiple-testing strategy: Family-wise FDR correction across predictor domains is statistically defensible but less stringent than omnibus correction across all 114 tests (19 regions × 6 families). This increases type I error risk relative to a single global threshold [77].
9. No behavioural or clinical correlates: The study provides structural reference data without cognitive, clinical, or functional outcomes, limiting direct translational implications [78].
10. Constrained functional form for age: Regional age associations were modelled using linear and quadratic terms only, selected *a priori* as part of a confirmatory, pre-registered design. This fixed low-order polynomial permits only a single symmetric inflexion and cannot capture more complex (e.g., bimodal or plateauing) trajectories. A post hoc comparison with natural cubic splines for the two regions with an *a priori* nonlinearity hypothesis (parietal lobe and white matter; Figure 5) supported the overall pattern of results but showed that turning-point estimates were model-dependent. As spline models were not evaluated for the remaining 17 regions, more complex nonlinear associations cannot be excluded. Future studies should re-estimate all regional age associations using GAMs or GAMLSS with penalised regression splines in larger, adequately powered samples.

We propose several extensions that build directly on these limitations. A prospective longitudinal follow-up of all participants after a 3-year interval, using the same scanner and acquisition protocol, represents the highest-priority extension of this work. This would yield true intra-individual atrophy rates, reduce survivorship bias, and enable prospective validation of the baseline volumetric predictors identified here.

Integration of the Vol2Brain structural metrics with the AgeRisk fMRI data acquired in the same session, specifically the Balloon Analogue Risk Task and delay discounting paradigms, would enable a direct within-participant structural-functional HAROLD test [79]. This would make it possible to examine whether individuals with reduced structural lateralisation in parietal and temporal regions also show reduced functional lateralisation during risky decision-making. Re-estimation of all 19 regional age associations using generalised additive models with penalised regression splines would relax polynomial curvature constraints and provide more precise inflexion estimates for WM and parietal associations without pre-specifying functional form.

Sex-stratified within-sample centile modelling using GAMLSS or Gaussian process regression [80,81,82] would extend the descriptive characterisation of regional volume variation toward a clinically useful framework for regional brain-age-gap estimation and early neurodegeneration screening. A comparison of Vol2Brain and FreeSurfer estimates across this age range, with particular attention to WM hyperintensity sensitivity in the oldest participants, would strengthen confidence in the pipeline-specific association estimates reported here. This would further support their translational use in lifespan clinical neuroimaging [83].

## 5. Conclusions

**Figure 19:**
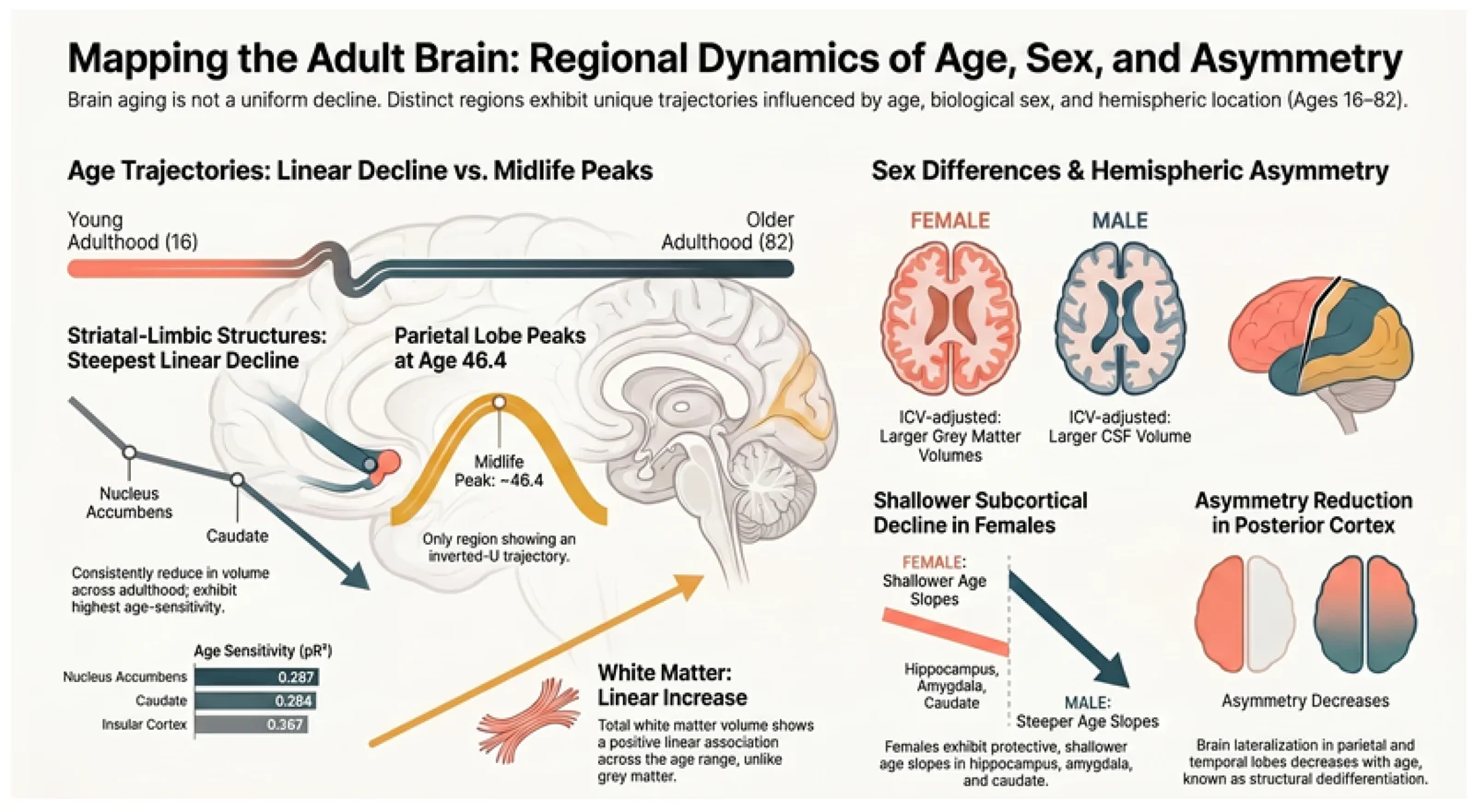
Establishing a normative structural reference for adult brain ageing. The precise characterisation of region-specific volumetric variation provides a disciplined cross-sectional baseline. This framework is essential for differentiating typical, healthy structural brain associations from the early morphological signatures of neurodegeneration.

This cross-sectional MRI analysis of 187 healthy adults investigated how brain volume varies with age, sex, and hemispheric asymmetry. Automated segmentation indicated that subcortical structures and specific cortical regions, such as the insula, exhibited significant negative linear cross-sectional associations with age. The parietal lobe was unique in demonstrating a nonlinear, inverted-U cross-sectional profile, whereas WM showed a positive linear association across the sampled age cohorts (16–82 years). As nonlinear estimates can depend on model specification, these findings should be interpreted alongside the spline sensitivity analyses rather than as definitive statements about underlying lifespan form. After adjusting for ICV, the study found that females generally had larger GM volumes and showed shallower negative age associations in several subcortical regions. The analysis also demonstrated that hemispheric asymmetry was lower at older ages, particularly in the posterior cortex, suggesting a structural pattern of age-related dedifferentiation. These findings (Table 9, Figure 19) offer a preliminary reference for distinguishing typical brain ageing from early signs of neurodegeneration within the studied cohort [84]. It should be noted, however, that all results were derived from the AgeRisk dataset, drawn from a specific Swiss community, and should be interpreted accordingly. Whether these patterns generalise across broader populations remains an open empirical question, and the present findings are best understood as community-specific observations rather than universal norms.

## Figures and Tables

**Table 1:** Pre-specified research hypotheses, predictions, and statistical tests.

Domain	Hypothesis Label	Directional Prediction	Deciding Test
Age association	H1a-i: Subcortical linear decline	Negative linear age association across subcortical regions	FDR-corrected p for linear age (m=19)
	H1a-ii: Parietal nonlinearity	Parietal lobe shows a nonlinear association	FDR-corrected p for quadratic age
	H1b: WM inverted-U	WM peaks in mid-adulthood before declining	FDR-corrected p for quadratic age
	H1c: Insular/striatal sensitivity	Accumbens, caudate, and insula show highest age $pR^{2}$	Rank order of linear age $pR^{2}$ across 19 regions
Sex differences	H2a: Female GM advantage	Greater ICV-adjusted GM volumes in females	FDR-corrected sex main effect in GM regions
	H2b: Male CSF advantage	Greater ICV-adjusted CSF volume in males	FDR-corrected sex main effect for CSF
	H2c: No sex effects in cerebellum/WM	Non-significant sex effects after ICV correction	FDR-corrected sex *p* >0.05 for cerebellum, WM
	H2d: Female-favouring interactions	Shallower cross-sectional age slopes in females for subcortical structures	FDR-corrected sex×age interaction across 19 regions
Hemispheric asymmetry	H3a: Population-level asymmetry	Directional lateralisation across bilateral structures	One-sample t-test vs. zero for each asymmetry index (AI), Bonferroni α=0.004
	H3b: Age-related asymmetry reduction	Negative age–AI correlation in posterior cortex; subcortical stability	Pearson *r* age–AI, Bonferroni (α=0.004) across 12 regions

**Table 2:** Worked verification that frontal lobe total volume equals the sum of its 17 constituent grey-matter gyri, shown for two participants selected at random. Values as stored in dataset.

	Sub-118	Sub-015
Gyrus ^ a ^	Male, 71 y	Female, 46 y
FRP	4.61	3.20
GRe	1.41	2.99
OpIFG	2.59	8.27
OrIFG	1.58	1.70
TrIFG	1.78	7.65
MFC	0.26	1.95
MFG	22.50	34.38
AOrG	1.22	3.22
LOrG	2.10	2.91
MOrG	1.67	7.01
POrG	1.67	5.97
PrG	17.57	28.15
MPrG	4.15	4.92
SCA	0.00	1.80
SFG	22.46	24.79
MSFG	6.02	9.53
SMC	6.60	9.04
Sum of 17 gyri	98.19	157.48
Reported “Frontal total volume” ^b^	98.20	157.48
Difference	0.01	0.00

^a^ FRP = Frontal Pole; GRe = Gyrus Rectus; OpIFG = Opercular part of the Inferior Frontal Gyrus; OrIFG = Orbital part of the Inferior Frontal Gyrus; TrIFG = Triangular part of the Inferior Frontal Gyrus; MFC = Medial Frontal Cortex; MFG = Middle Frontal Gyrus; AOrG = Anterior Orbital Gyrus; LOrG = Lateral Orbital Gyrus; MOrG = Medial Orbital Gyrus; POrG = Posterior Orbital Gyrus; PrG = Precentral Gyrus; MPrG = Precentral Gyrus Medial Segment; SCA = Subcallosal Area; SFG = Superior Frontal Gyrus; MSFG = Superior Frontal Gyrus Medial Segment; SMC = Supplementary Motor Cortex. ^b^ Column “Frontal total volume cm^3^” in the raw Vol2Brain export, i.e., the value used directly in all age and sex × age models reported in Section 3.2.

**Table 3:** Decade-stratified regional brain volumes (cm^3^) in the present sample.

Age Group	16–29 yr		30–39 yr		40–49 yr		50–59 yr		60–69 yr		70+ yr	
Sex	M	F	M	F	M	F	M	F	M	F	M	F
n	27	29	13	15	9	12	11	13	15	18	15	10
Hippocampus	7.43 ± 0.95	7.06 ± 0.84	7.49 ± 0.89	7.12 ± 0.75	7.21 ± 1.19	6.77 ± 0.78	6.87 ± 1.07	6.51 ± 2.34	6.49 ± 2.12	7.09 ± 0.8	5.6 ± 1.91	6.33 ± 2.25
Amygdala	2.02 ± 0.28	1.77 ± 0.29	2.02 ± 0.18	1.77 ± 0.22	1.8 ± 0.31	1.64 ± 0.22	1.72 ± 0.3	1.53 ± 0.56	1.63 ± 0.51	1.72 ± 0.21	1.24 ± 0.51	1.32 ± 0.41
Caudate	8.17 ± 1.09	7.3 ± 0.57	7.56 ± 1.13	6.95 ± 0.89	6.93 ± 1.05	6.83 ± 0.86	7.19 ± 0.96	6.18 ± 1.86	7.05 ± 0.87	6.39 ± 0.86	5.69 ± 1.65	5.94 ± 1.53
Putamen	7.81 ± 1.92	6.87 ± 1.36	7.54 ± 1.91	6.95 ± 1.91	7.2 ± 1.81	6.19 ± 1.15	6.74 ± 1.33	5.02 ± 2.29	6.55 ± 1.7	6.44 ± 1.27	4.91 ± 2.25	5.39 ± 2.24
Accumbens	0.64 ± 0.11	0.55 ± 0.11	0.584 ± 0.147	0.53 ± 0.13	0.51 ± 0.19	0.43 ± 0.07	0.55 ± 0.09	0.39 ± 0.17	0.45 ± 0.12	0.45 ± 0.11	0.3 ± 0.213	0.37 ± 0.16
Thalamus	5.34 ± 2.3	5.92 ± 3.06	5.17 ± 1.29	7.0 ± 3.86	4.03 ± 0.72	4.4 ± 1.44	5.74 ± 3.81	4.22 ± 2.69	4.84 ± 3.1	5.9 ± 2.52	3.13 ± 1.14	4.62 ± 3.24
Pallidum	2.06 ± 0.46	2.13 ± 0.54	2.201 ± 0.39	2.24 ± 0.6	1.76 ± 0.39	1.86 ± 0.36	2.27 ± 0.65	1.95 ± 0.6	2.26 ± 0.58	2.03 ± 0.57	2.28 ± 0.72	2.06 ± 0.83
Subcortical GM	34.2 ± 5.6	32.2 ± 5.5	33.2 ± 4.8	33.1 ± 2.2	30.0 ± 4.8	28.6 ± 3.1	31.7 ± 5.8	26.3 ± 9.7	29.8 ± 6.5	30.6 ± 5.0	23.6 ± 7.3	26.5 ± 9.2
Cortical GM	493 ± 65	497 ± 64	541 ± 79	482 ± 55	499 ± 38	473 ± 46	520 ± 43	493 ± 82	506 ± 57	481 ± 46	495 ± 90	456 ± 109
Total GM	628 ± 72	626 ± 71	674 ± 93	610 ± 66	622 ± 47	592 ± 55	651 ± 47	608 ± 108	622 ± 70	603 ± 56	599 ± 112	565 ± 139
WM	395 ± 47	366 ± 33	432 ± 51	370 ± 51	421 ± 32	384 ± 33	440 ± 58	404 ± 39	414 ± 31	363 ± 34	422 ± 64	377 ± 72
CSF	337 ± 64	269 ± 53	308 ± 42	233 ± 58	313 ± 62	256 ± 48	303 ± 61	264 ± 61	316 ± 67	246 ± 38	307 ± 69	251 ± 73
Cerebellum	139.4 ± 12.8	126.8 ± 9.7	138.0 ± 16.8	124.8 ± 12.0	135.8 ± 9.8	121.3 ± 7.4	137.3 ± 11.3	121.4 ± 18.0	124.8 ± 16.8	117.3 ± 10.4	121.3 ± 19.5	110.5 ± 27.9
Cerebrum	876 ± 96	858 ± 91	960 ± 118	847 ± 97	900 ± 71	848 ± 64	946 ± 89	884 ± 117	904 ± 72	841 ± 75	893 ± 140	825 ± 174
Temporal Lobe	107.8 ± 12.9	104.6 ± 11.2	110.7 ± 16.3	99.8 ± 10.7	102.5 ± 7.6	94.9 ± 10.1	104.7 ± 11.4	97.5 ± 17.6	101.3 ± 12.2	97.7 ± 8.5	97.1 ± 20.4	89.0 ± 22.4
Frontal Lobe	125.4 ± 32.4	139.7 ± 28.5	150.7 ± 29.8	139.2 ± 24.1	141.4 ± 23.4	142.9 ± 22.3	153.7 ± 28.9	149.9 ± 26.8	153.1 ± 20.1	147.5 ± 22.6	163.6 ± 28.9	147.2 ± 32.4
Parietal Lobe	99.6 ± 13.6	100.0 ± 15.6	114.8 ± 17.8	100.8 ± 10.9	104.1 ± 8.4	99.0 ± 9.9	109.0 ± 9.1	103.8 ± 15.8	103.7 ± 14.2	97.8 ± 11.3	97.2 ± 16.8	92.1 ± 23.9
Occipital Lobe	77.5 ± 6.2	75.5 ± 8.8	80.4 ± 8.9	69.4 ± 8.1	72.7 ± 7.8	67.7 ± 6.9	75.9 ± 5.6	70.7 ± 12.6	72.0 ± 8.5	68.8 ± 5.4	67.9 ± 14.5	64.0 ± 16.0
Insula	35.4 ± 5.3	32.0 ± 3.4	35.4 ± 4.4	29.5 ± 3.2	31.2 ± 1.8	28.0 ± 3.3	31.9 ± 3.1	28.9 ± 2.8	31.4 ± 5.4	28.0 ± 2.2	27.7 ± 6.8	25.2 ± 3.6

**Table 4:** Condensed list of FDR-significant predictors across regions. Full regression coefficients for all predictors and regions are in Supplementary Table S1.

Region	Predictor	Direction/β (95% CI)	$p_{\mathrm{FDR}}$/*p*	${\mathrm{pR}}^{2}$ (Unique Variance)
Hippocampus	Age (linear)	Negative; β=−0.016; 95% CI [−0.024,−0.007]	$p_{\mathrm{FDR}}<$ 0.001	0.070
Hippocampus	Sex (female > male)	β=+0.79; 95% CI [+0.424,+1.152]	$p_{\mathrm{FDR}}<$ 0.001	0.090
Amygdala	Age (linear)	Negative; full β in Table S1	$p_{\mathrm{FDR}}<$ 0.05	(see Table S1)
Caudate	Age (linear)	Negative; full β in Table S1	$p_{\mathrm{FDR}}<$ 0.05	(see Table S1)
Putamen	Age (linear)	Negative; full β in Table S1	$p_{\mathrm{FDR}}<$ 0.05	(see Table S1)
Nucleus accumbens	Age (linear)	Negative; full β in Table S1	$p_{\mathrm{FDR}}<$ 0.05	(see Table S1)
Insular cortex	Age (linear)	Negative; full β in Table S1	$p_{\mathrm{FDR}}<$ 0.05	(see Table S1)
Temporal lobe	Age (linear)	Negative; full β in Table S1	$p_{\mathrm{FDR}}<$ 0.05	(see Table S1)
Occipital lobe	Age (linear)	Negative; full β in Table S1	$p_{\mathrm{FDR}}<$ 0.05	(see Table S1)
Parietal lobe	Age^2^ (quadratic)	Inverted-U; vertex ≈ midlife; full β in Table S1	$p_{\mathrm{FDR}}=$ 0.008	(see Table S1)
WM (total)	Age (linear)	Positive linear association and no FDR-significant quadratic term; full β in Table S1	$p_{\mathrm{FDR}}<$ 0.05	(see Table S1)
Cerebrospinal fluid (CSF)	Sex (male > female)	Male > female (ICV-adjusted); full β in Table S1	$p_{\mathrm{FDR}}<$ 0.05	(see Table S1)
Asymmetry: Parietal AI	Age → AI	Negative (reduced asymmetry with age)	p< 0.001	(see Table S1)

**Table 5:** Sex × age interaction coefficients for 19 brain regions. FDR-significant rows are highlighted.

Region	Male β	Female β	$\beta_{\mathrm{int}}$	SE	*p* (Raw)	$p_{\mathrm{FDR}}$
	(cm^3^/yr)	(cm^3^/yr)				
Hippocampus	−0.028	−0.004	**+0.024**	0.008	0.005	0.029 *
Amygdala	−0.013	−0.004	**+0.008**	0.002	0.001	0.007 **
Caudate	−0.039	−0.021	**+0.018**	0.007	0.009	0.034 *
Putamen	−0.047	−0.023	+0.024	0.013	0.063	0.150
Accumbens	−0.006	−0.003	**+0.003**	0.001	0.002	0.019 *
Thalamus ^†^	−0.029	−0.020	+0.009	0.021	0.677	0.756
Pallidum	+0.005	−0.001	−0.006	0.004	0.116	0.244
Subcortical GM	−0.160	−0.077	+0.083	0.043	0.054	0.150
Cortical GM	+0.235	−0.120	−0.355	0.301	0.239	0.349
Total GM	−0.240	−0.341	−0.102	0.351	0.773	0.815
WM	+0.726	+0.448	−0.278	0.234	0.235	0.349
CSF	−0.459	−0.106	+0.354	0.400	0.377	0.478
Cerebellum	−0.273	−0.179	+0.095	0.071	0.186	0.349
Cerebrum	+0.782	+0.292	−0.491	0.387	0.207	0.349
Temporal Lobe	−0.168	−0.155	+0.014	0.062	0.828	0.828
Frontal Lobe	+0.749	+0.301	**−0.448**	0.168	0.009	0.034 *
Parietal Lobe	+0.038	+0.001	−0.037	0.070	0.594	0.706
Occipital Lobe	−0.152	−0.112	+0.040	0.040	0.321	0.436
Insula	−0.127	−0.079	+0.047	0.025	0.060	0.150

* $p_{\mathrm{FDR}}<0.05$; ** $p_{\mathrm{FDR}}<0.01$. Bold $\beta_{\mathrm{int}}$ values indicate FDR-significant sex × age interaction coefficients. Grey shading likewise identifies rows with FDR-significant interaction terms. ^†^ Thalamic absolute-volume interaction shown for completeness but excluded from primary H2d interpretation (CV =53.4%); thalamic AI retained for H3.

**Table 6:** Population-level hemispheric AIs across 12 confirmatory bilateral structures.

Region	N (Fail)	$\bar{R}$ (cm^3^)	$\bar{L}$ (cm^3^)	Mean AI (%)	SD	Median [IQR]	Boot. 95% CI	Raw Test and *p*	Adj. *p*	Verdict
*Left-hemisphere dominant (AI < 0)*										
Hippocampus	184 (f = 3)	3.33	3.65	−10.59	19.37	−6.83 {[−13.99, −0.27]}	[−13.46, −7.89]	Wilcoxon, <0.001	<0.001	✓
Insula	187 (f = 0)	14.68	16.19	−10.15	8.40	−9.38 {[−14.24, −4.42]}	[−11.39, −9.03]	Wilcoxon, <0.001	<0.001	✓
Amygdala	184 (f = 3)	0.83	0.90	−10.01	18.92	−8.21 {[−15.70, −1.12]}	[−12.77, −7.31]	Wilcoxon, <0.001	<0.001	✓
Thalamus ^a^	187 (f = 0)	2.47	2.70	−9.39	11.38	−9.16 {[−16.62, −1.78]}	[−11.01, −7.80]	Wilcoxon, <0.001	<0.001	✓
Pallidum	187 (f = 0)	1.01	1.09	−7.70	22.67	−8.89 {[−19.28, +5.71]}	[−10.98, −4.41]	Wilcoxon, <0.001	<0.001	✓
Putamen	185 (f = 2)	3.23	3.42	−6.00	14.47	−4.47 {[−14.09, +2.99]}	[−8.15, −3.94]	Wilcoxon, <0.001	<0.001	✓
*Right-hemisphere dominant (AI > 0)*										
Accumbens	180 (f = 7)	0.25	0.25	+0.72	18.03	+0.00 {[−10.91, +9.52]}	[−1.84, +3.40]	Wilcoxon, 0.819	0.592	✗ ns
Temporal lobe	187 (f = 0)	51.12	50.41	+1.33	6.67	+0.44 {[−3.08, +5.92]}	[+0.39, +2.31]	Wilcoxon, 0.020	0.002	✓
Frontal lobe	187 (f = 0)	72.75	71.59	+1.62	5.14	+1.59 {[−1.19, +4.67]}	[+0.88, +2.35]	*t*-test, <0.001	<0.001	✓
Cerebrum	187 (f = 0)	448.74	429.52	+4.43	2.70	+4.15 {[+2.37, +6.10]}	[+4.05, +4.83]	Wilcoxon, <0.001	<0.001	✓
Caudate	187 (f = 0)	3.57	3.40	+4.92	6.45	+4.71 {[+2.26, +7.38]}	[+3.98, +5.80]	Wilcoxon, <0.001	<0.001	✓
Parietal lobe	187 (f = 0)	52.14	49.27	+5.77	6.66	+5.41 {[+1.38, +10.38]}	[+4.81, +6.73]	*t*-test, <0.001	<0.001	✓

^a^ Thalamic AI was retained because bilateral volumes were highly correlated (r=0.973), whereas AI was not associated with volume (p=0.066) or SNR (p=0.91; Section 3.4). $\bar{R}$/$\bar{L}$ = mean right/left volume (cm^3^); positive/negative AI = right/left dominance. Adj. *p* = age- and ICV-adjusted OLS intercept *p*-value. Wilcoxon = signed-rank test; otherwise, one-sample *t*-test. *f* = excluded segmentation failures. Bonferroni α=0.004. ✓ = significant; ✗ ns = not significant.

**Table 7:** Age-related asymmetry reduction across 12 confirmatory regions. Thalamus absolute volume was excluded from H1/H2 (CV =53.4%) but retained here for AI because the within-subject metric was stable (bilateral r=0.97, 0 failures, SD=11.4%).

	Region	Partial *r*	95% CI	Raw *p*	Quadratic *p*	Verdict
Cortical & Global	Parietal lobe	−0.534	[−0.635, −0.413]	<0.001	0.270	✓
	Temporal lobe	−0.513	[−0.548, −0.288]	<0.001	0.435	✓
	Cerebrum	−0.318	[−0.444, −0.176]	<0.001	0.390	✓
	Insula	−0.252	[−0.385, −0.108]	<0.001	0.393	✓
	Frontal lobe	−0.173	[−0.294, −0.007]	0.034	0.866	✓
Subcortical	Amygdala	−0.242	[−0.376, −0.096]	<0.001	0.069	✓
	Hippocampus	−0.207	[−0.344, −0.060]	0.005	0.376	✗ ns
	Caudate	+0.011	[+0.052, +0.333]	0.016	0.679	✗ ns
	Putamen	−0.058	[−0.204, +0.091]	0.435	0.102	✗ ns
	Accumbens	−0.040	[−0.188, +0.110]	0.579	0.093	✗ ns
	Pallidum	+0.014	[−0.193, +0.158]	0.889	0.229	✗ ns
	Thalamus	+0.010	[−0.139, +0.158]	0.889	0.229	✗ ns

Partial *r* = age–AI Pearson correlation controlling for sex and ICV; 95% CI = bootstrap CI (10,000 iterations); raw *p* = two-tailed *t*-test; quadratic *p* = *p*-value for the age^2^ term in AI ∼ age^2^ regression. ✓ = significant linear age effect (p<0.05); ✗ ns = non-significant.

**Table 8:** Sex differences in asymmetry indices across the 12 confirmatory bilateral regions (H3c). Thalamus retained for AI despite absolute-volume exclusion (CV =53.4%); AI stable (bilateral r=0.97, 0 failures, SD=11.4%).

Region	Sex β	SE	*t*	Raw *p*	Bonf. *p*	Verdict
**Cortical & Global**						
Cerebrum GM	+0.17	0.44	−0.39	0.699	1.000	✗ ns
Frontal lobe	−0.54	0.85	−0.64	0.522	1.000	✗ ns
Temporal lobe	+1.44	0.95	−1.51	0.133	1.000	✗ ns
Parietal lobe	+0.43	0.94	−0.46	0.647	1.000	✗ ns
Occipital lobe ^†^	+1.77	1.17	−1.51	0.133	1.000	✗ ns
Limbic cortex ^†^	−0.46	0.95	−0.48	0.630	1.000	✗ ns
**Subcortical**						
Hippocampus	−8.62	3.23	−2.67	0.008	1.000	✗ ns
Amygdala	−4.92	3.12	−1.58	0.117	1.000	✗ ns
Thalamus	−0.18	1.88	−0.10	0.922	1.000	✗ ns
Caudate	−1.02	1.03	−0.99	0.325	1.000	✗ ns
Putamen	−0.82	2.38	−0.35	0.729	1.000	✗ ns
Cerebellum GM ^†^	−1.29	1.21	−1.07	.287	1.000	✗ ns

*Note.*β = unstandardised regression coefficient for Sex (male = 1), representing the mean AI difference (Male − Female). Model controls for mean-centred linear age, quadratic age, and ICV. Bonf. *p* reflects strict Bonferroni correction for 12 tests (α=0.004). ✗ ns = not significant. ^†^ Region not in primary pre-specified set. Thalamus absolute volume excluded from H1/H2 due to high between-subject CV (53.4%) but retained for AI because AI is a within-subject ratio with demonstrated stability: bilateral r=0.97, 0/187 failures, 0/187|AI|>100%, AI SD=11.4%.

**Table 9:** Hypothesis verdicts and effect sizes.

Domain	Hypothesis and Verdict	Prediction	Key Evidence
Age association	H1a-i: Subcortical linear decline (Supported)	Negative linear age (9 regions)	FDR-significant negative linear age associations in multiple subcortical regions (hippocampus $pR^{2}=0.070$, $\beta =-0.016{\mathrm{cm}}^{3}/\mathrm{yr}$; amygdala, caudate, putamen, nucleus accumbens; all $p_{\mathrm{FDR}}<0.05$). Thalamus absolute volume nominally significant ($p_{\mathrm{FDR}}=0.026$) but designated preliminary and excluded from primary volume interpretation due to absolute-volume CV =53.4%; thalamic AI retained separately under H3 due to within-subject stability (bilateral r=0.97, 0 failures, SD=11.4%).
	H1a-ii: Parietal nonlinearity (Supported)	Parietal lobe shows a nonlinear association	The parietal lobe was the sole region (1 of 19) with a significant nonlinear association ($p_{\mathrm{FDR}}=0.008$, $pR^{2}=0.067$): inverted-U peaking at 46.4 yr (bootstrap 95% CI: 40.6 yr to 51.3 yr).
	H1b: WM inverted-U (Not Supported)	Quadratic peak midlife	Positive linear association; $\beta_{2}$ non-significant. Linear $\beta =0.587{\mathrm{cm}}^{3}/\mathrm{yr}$ ($p_{\mathrm{FDR}}<0.001$, $pR^{2}=0.121$); quadratic non-significant ($p_{\mathrm{FDR}}=0.174$). Estimated inflexion: ≈61.5 yr, but did not survive FDR ($p_{\mathrm{FDR}}=0.173$).
	H1c: Insular/striatal sensitivity (Supported)	Highest age $pR^{2}$	Accumbens (1st, age $pR^{2}=0.287$), caudate (2nd, $pR^{2}=0.284$), insula (3rd, $pR^{2}=0.267$) in age-sensitivity.
Sex differences	H2a: Female GM advantage (Supported)	Greater ICV-adj. GM	Post-ICV adjustment, females were larger in 10 of 19 regions (all $p_{\mathrm{FDR}}<0.05$). Total GM $\beta =42.7{\mathrm{cm}}^{3}$ ($p_{\mathrm{FDR}}<0.01$, $pR^{2}=0.149$); Cortical GM: $\beta =32.8{\mathrm{cm}}^{3}$, $pR^{2}=0.123$; Hippocampus: $\beta =0.79{\mathrm{cm}}^{3}$, $pR^{2}=0.090$; Total/cortical GM, hippocampus significant (all $p_{\mathrm{FDR}}<0.01$).
	H2b: Male CSF advantage (Supported)	Greater ICV-adj. CSF	$\beta_{\mathrm{male}}=36.2{\mathrm{cm}}^{3}$ ($p_{\mathrm{FDR}}<0.001$, $pR^{2}=0.088$). Male > female, $p_{\mathrm{FDR}}<0.001$.
	H2c: No cerebellum/WM sex effects (Supported)	p>0.05 after ICV	No FDR-significant sex effects (Cerebellum $p_{\mathrm{FDR}}=0.752$; WM $p_{\mathrm{FDR}}=0.301$; both $p_{\mathrm{FDR}}>0.50$ after ICV correction).
Hemispheric Asymmetry	H2d: Female-favouring interactions (subcortical associations) (Partially Supported)	Shallower subcortical slopes	4 of 9 subcortical regions showed FDR-significant female-protective interactions (hippocampus, amygdala, accumbens, caudate). Hippocampus: $\beta_{\mathrm{int}}=0.024{\mathrm{cm}}^{3}/\mathrm{yr}$, $p_{\mathrm{FDR}}=0.029$, $pR^{2}=0.044$; Amygdala: $\beta_{\mathrm{int}}=0.008{\mathrm{cm}}^{3}/\mathrm{yr}$, $p_{\mathrm{FDR}}=0.007$, $pR^{2}=0.068$; Caudate: $p_{\mathrm{FDR}}=0.034$; Accumbens: $p_{\mathrm{FDR}}=0.019$. Interactions modest and region-specific.
	H3a: Population-level asymmetry (Supported)	Directional AI ≠ 0 (12 regions)	Directional lateralisation confirmed in 11 of 12 pre-specified bilateral structures (all p<0.004 Bonferroni); accumbens ns/exception (p=0.819). Exploratory: WM mean AI = 10.71%, p<0.001; Cerebellum mean AI = 4.09%, p<0.001.
	H3b: Age-related reduction (Partially Supported)	Posterior cortex r<0; subcortical stable	Age-related AI reduction confined to the posterior cortex. Most subcortical structures are stable (all $p_{\mathrm{FDR}}>0.10$ except amygdala: r=−0.242, p<0.001).

## Data Availability

The original contributions presented in this study are included in the article/Supplementary Materials. Further inquiries can be directed to the corresponding author.

## Supplementary Materials

The following supporting information can be downloaded at: https://www.mdpi.com/article/10.3390/life16081356/s1, Supplementary Table S1: Complete statistical table (PDF).

## Abbreviations

The following abbreviations are used in this manuscript:

AI	Asymmetry Index
ANCOVA	Analysis of Covariance
BIDS	Brain Imaging Data Structure
BET	Brain Extraction Tool
CI	Confidence Interval
CortGM	Cortical Grey Matter
CSF	Cerebrospinal Fluid
CV	Coefficient of Variation
DTI	Diffusion Tensor Imaging
FDR	False Discovery Rate
fMRI	Functional Magnetic Resonance Imaging
FSL	FMRIB Software Library
GAMLSS	Generalised Additive Models for Location, Scale and Shape
GM	Grey Matter
HAROLD	Hemispheric Asymmetry Reduction in Older Adults
ICV	Intracranial Volume
IQR	Interquartile Range
MNI	Montreal Neurological Institute
MRI	Magnetic Resonance Imaging
NAcc	Nucleus Accumbens
OLS	Ordinary Least Squares
OSF	Open Science Framework
PET	Positron-emission-tomography
$p_{\mathrm{FDR}}$	Benjamini–Hochberg false discovery rate corrected *p*-value
$pR^{2}$	Semi-partial $R^{2}$ (unique variance explained by a single predictor)
*r*	Pearson partial correlation coefficient
$R_{\mathrm{adj}}^{2}$	Adjusted $R^{2}$ (model-level goodness-of-fit statistic)
SD	Standard Deviation
SE	Standard Error
SNR	Signal-to-Noise Ratio
SubGM	Subcortical Grey Matter
T1	T1-weighted MRI contrast
TotGM	Total Grey Matter
WM	White Matter
