# Supplementary material for: Regional Brain Volume Variation Across Adulthood: A Cross-Sectional MRI Analysis of Age, Sex, and Hemispheric Asymmetry
Source: Life (Basel). 2026 Aug 18;16(8):1356. doi: 10.3390/life16081356 (PMC13514872; doi:10.3390/life16081356)
Supplement: Supplementary file 1 [file life-16-01356-s001.zip › life-4410284-supplementary.pdf]

Supplementary Materials

Supplementary Table S1: Complete statistical table.

Table S1. Association of age, sex, ICV and SNR with regional brain volumes.

Region and Mean ± SD (cm^3)	Predictor	Standardised β	Unstandardised β	95% CI	p (raw)	p_{FDR}	pR^2	Vertex Age (yr)	Association
**Hippocampus** 6.9 ± 1.4; R^2_{adj} = 0.379	Age (linear)	-0.215	-0.016	[-0.024, -0.007]	<0.001	<0.001†	0.070	5.8	Linear ↓
	Age^2	-0.043	-0.00020	[-0.00074, +0.00034]	0.466	0.806	0.003		
	Sex	-0.278	+0.79	[+0.424, +1.152]	<0.001	<0.001†	0.090		
	ICV	+0.641	+0.0064	[+0.0050, +0.0077]	<0.001	<0.001†	0.334		
	SNR	+0.059	+0.00105	[-0.00103, +0.00313]	0.322	0.762	0.005		
**Amygdala** 1.7 ± 0.4; R^2_{adj} = 0.404	Age (linear)	-0.401	-0.009	[-0.011, -0.006]	<0.001	<0.001†	0.215	16.2	Linear ↓
	Age^2	-0.110	-0.00015	[-0.00030, +0.00001]	0.059	0.266	0.020		
	Sex	-0.069	+0.06	[-0.046, +0.158]	0.282	0.383	0.006		
	ICV	+0.484	+0.0014	[+0.0010, +0.0017]	<0.001	<0.001†	0.229		
	SNR	+0.110	+0.00056	[-0.00002, +0.00114]	0.060	0.575	0.019		
**Caudate** 7.0 ± 1.3; R^2_{adj} = 0.499	Age (linear)	-0.447	-0.030	[-0.037, -0.023]	<0.001	<0.001†	0.284	125‡	Linear ↓
	Age^2	+0.044	+0.00019	[-0.00026, +0.00063]	0.407	0.774	0.004		
	Sex	-0.042	+0.11	[-0.191, +0.407]	0.480	0.608	0.003		
	ICV	+0.565	+0.0051	[+0.0040, +0.0062]	<0.001	<0.001†	0.325		
	SNR	+0.074	+0.00121	[-0.00049, +0.00292]	0.165	0.612	0.011		
**Putamen** 6.6 ± 1.9; R^2_{adj} = 0.244	Age (linear)	-0.345	-0.035	[-0.048, -0.022]	<0.001	<0.001†	0.136	-378‡	Linear ↓
	Age^2	-0.006	-0.00004	[-0.00086, +0.00078]	0.922	0.943	<0.001		
	Sex	+0.001	-0.01	[-0.554, +0.543]	0.984	0.984	<0.001		
	ICV	+0.367	+0.0050	[+0.0030, +0.0069]	<0.001	<0.001†	0.119		
	SNR	+0.128	+0.00311	[-0.00002, +0.00624]	0.053	0.575	0.021		
**Accumbens** 0.5 ± 0.2; R^2_{adj} = 0.425	Age (linear)	-0.481	-0.004	[-0.005, -0.003]	<0.001	<0.001†	0.287	-119‡	Linear ↓
	Age^2	-0.023	-0.00001	[-0.00007, +0.00005]	0.686	0.895	<0.001		
	Sex	-0.031	+0.01	[-0.030, +0.050]	0.628	0.746	0.001		
	ICV	+0.443	+0.0005	[+0.0004, +0.0006]	<0.001	<0.001†	0.205		
	SNR	+0.086	+0.00018	[-0.00005, +0.00041]	0.136	0.612	0.012		
**Thalamus** 5.2 ± 2.8; R^2_{adj} = 0.055	Age (linear)	-0.170	-0.025	[-0.045, -0.004]	0.019	0.026*	0.030	-28‡	Linear ↓
	Age^2	-0.018	-0.00017	[-0.00147, +0.00113]	0.802	0.943	<0.001		
	Sex	-0.211	+1.16	[+0.289, +2.038]	0.010	0.019*	0.036		
	ICV	+0.185	+0.0036	[+0.0004, +0.0067]	0.027	0.027*	0.027		
	SNR	+0.030	+0.00103	[-0.00396, +0.00602]	0.686	0.825	<0.001		

Continued on next page

Region and Mean ± SD (cm^3)	Predictor	Standardised β	β	95% CI	p (raw)	p_{FDR}	pR^2	Vertex Age (yr)	Association
**Pallidum** 2.1 ± 0.6; R^2_{adj} = 0.209	Age (linear)	+0.065	+0.002	[-0.002, +0.006]	0.310	0.346	0.006	40.8	Stable (ns)
	Age^2	+0.121	+0.00023	[-0.00002, +0.00047]	0.070	0.266	0.018		
	Sex	-0.181	+0.20	[+0.041, +0.368]	0.015	0.024*	0.032		
	ICV	+0.545	+0.0022	[+0.0016, +0.0027]	<0.001	<0.001†	0.221		
	SNR	+0.060	+0.00043	[-0.00050, +0.00136]	0.370	0.762	0.004		
**Subcortical GM** 30.5 ± 6.9; R^2_{adj} = 0.323	Age (linear)	-0.332	-0.118	[-0.161, -0.076]	<0.001	<0.001†	0.140	-550‡	Linear ↓
	Age^2	-0.004	-0.00010	[-0.00283, +0.00263]	0.943	0.943	<0.001		
	Sex	-0.170	+2.33	[+0.496, +4.167]	0.014	0.024*	0.033		
	ICV	+0.515	+0.0247	[+0.0181, +0.0313]	<0.001	<0.001†	0.229		
	SNR	+0.089	+0.00770	[-0.00277, +0.01817]	0.151	0.612	0.011		
**Cortical GM** 494.8 ± 67.2; R^2_{adj} = 0.659	Age (linear)	+0.017	+0.057	[-0.239, +0.354]	0.705	0.705	<0.001	53.0	Stable (ns)
	Age^2	-0.016	-0.00366	[-0.02268, +0.01537]	0.707	0.895	<0.001		
	Sex	-0.245	+32.84	[+20.063, +45.624]	<0.001	<0.001†	0.123		
	ICV	+0.900	+0.4230	[+0.3771, +0.4690]	<0.001	<0.001†	0.643		
	SNR	+0.008	+0.00705	[-0.06585, +0.07996]	0.850	0.850	<0.001		
**Total GM** 618.2 ± 81.4; R^2_{adj} = 0.686	Age (linear)	-0.068	-0.291	[-0.635, +0.054]	0.100	0.127	0.015	16.1	Stable (ns)
	Age^2	-0.019	-0.00501	[-0.02711, +0.01710]	0.658	0.895	0.001		
	Sex	-0.263	+42.67	[+27.820, +57.516]	<0.001	<0.001†	0.149		
	ICV	+0.912	+0.5193	[+0.4659, +0.5727]	<0.001	<0.001†	0.668		
	SNR	+0.023	+0.02321	[-0.06149, +0.10791]	0.592	0.825	0.002		
**WM** 394.5 ± 51.2; R^2_{adj} = 0.645	Age (linear)	+0.223	+0.587	[+0.356, +0.818]	<0.001	<0.001†	0.121	61.5	Linear
	Age^2	-0.106	-0.01796	[-0.03275, -0.00318]	0.018	0.173	0.030		
	Sex	+0.063	-6.43	[-16.362, +3.497]	0.206	0.301	0.009		
	ICV	+0.746	+0.2669	[+0.2312, +0.3026]	<0.001	<0.001†	0.543		
	SNR	-0.015	-0.00968	[-0.06632, +0.04697]	0.738	0.825	<0.001		
**CSF** 285.2 ± 65.8; R^2_{adj} = 0.374	Age (linear)	-0.085	-0.282	[-0.676, +0.112]	0.162	0.193	0.011	51.0	Stable (ns)
	Age^2	+0.110	+0.02397	[-0.00126, +0.04921]	0.064	0.266	0.019		
	Sex	+0.275	-36.16	[-53.107, -19.205]	<0.001	<0.001†	0.088		
	ICV	+0.433	+0.1994	[+0.1384, +0.2603]	<0.001	<0.001†	0.185		
	SNR	-0.017	-0.01372	[-0.11042, +0.08297]	0.781	0.825	<0.001		
**Cerebellum** 127.2 ± 16.5; R^2_{adj} = 0.680	Age (linear)	-0.322	-0.226	[-0.296, -0.156]	<0.001	<0.001†	0.180	-31‡	Linear ↓
	Age^2	-0.028	-0.00148	[-0.00599, +0.00302]	0.519	0.822	0.002		
	Sex	-0.274	-0.65	[-3.682, +2.376]	0.673	0.752	<0.001		
	ICV	+0.746	+0.0852	[+0.0743, +0.0961]	<0.001	<0.001†	0.565		
	SNR	+0.049	-0.01036	[-0.02764, +0.00692]	0.242	0.656	0.008		

Continued on next page

Region and Mean ± SD (cm^3)	Predictor	Standardised β	β	95% CI	p (raw)	p_{FDR}	pR^2	Vertex Age (yr)	Association
**Cerebrum**<br>878.3 ± 105.2; R^2_{adj} = 0.769	Age (linear)	+0.100	+0.537	[+0.154, +0.919]	0.006	0.009**	0.040	57.7	Linear ↑
	Age^2	-0.062	-0.02145	[-0.04594, +0.00304]	0.088	0.278	0.016		
	Sex	-0.171	+35.84	[+19.387, +52.290]	<0.001	<0.001†	0.091		
	ICV	+0.943	+0.6943	[+0.6352, +0.7535]	<0.001	<0.001†	0.745		
	SNR	+0.017	+0.02250	[-0.07135, +0.11635]	0.639	0.825	0.001		
**Temporal Lobe**<br>101.5 ± 14.3; R^2_{adj} = 0.680	Age (linear)	-0.218	-0.162	[-0.223, -0.100]	<0.001	<0.001†	0.128		Linear ↓
	Age^2	+0.056	+0.00264	[-0.00129, +0.00658]	0.190	0.515	0.009	—	
	Sex	-0.190	+5.45	[+2.809, +8.092]	<0.001	<0.001†	0.083		
	ICV	+0.865	+0.0867	[+0.0772, +0.0962]	<0.001	<0.001†	0.639		
	SNR	+0.036	+0.00647	[-0.00860, +0.02155]	0.401	0.762	0.004		
**Frontal Lobe**<br>144.3 ± 28.5; R^2_{adj} = 0.385	Age (linear)	+0.355	+0.525	[+0.356, +0.693]	<0.001	<0.001†	0.170	429‡	Linear ↑
	Age^2	-0.007	-0.00068	[-0.01151, +0.01014]	0.901	0.943	<0.001		
	Sex	-0.274	+15.58	[+8.309, +22.848]	<0.001	<0.001†	0.089	—	—
	ICV	+0.624	+0.1243	[+0.0982, +0.1504]	<0.001	<0.001†	0.324		
	SNR	-0.031	-0.01099	[-0.05246, +0.03047]	0.604	0.825	0.002		
**Parietal Lobe**<br>101.4 ± 14.9; R^2_{adj} = 0.632	Age (linear)	+0.029	+0.020	[-0.049, +0.088]	0.574	0.606	0.002	46.4◇	Quadratic
	Age^2	-0.164	-0.00807	[-0.01246, -0.00368]	<0.001	0.008**	0.067		
	Sex	-0.231	+6.88	[+3.938, +9.833]	<0.001	<0.001†	0.104		
	ICV	+0.855	+0.0893	[+0.0787, +0.0998]	<0.001	<0.001†	0.601		
	SNR	+0.060	+0.01120	[-0.00561, +0.02801]	0.193	0.612	0.009		
**Occipital Lobe**<br>72.6 ± 10.0; R^2_{adj} = 0.725	Age (linear)	-0.255	-0.132	[-0.172, -0.092]	<0.001	<0.001†	0.191	103.6	Linear ↓
	Age^2	+0.034	+0.00113	[-0.00141, +0.00367]	0.385	0.774	0.004		
	Sex	-0.178	+3.55	[+1.842, +5.257]	<0.001	<0.001†	0.084		
	ICV	+0.869	+0.0608	[+0.0547, +0.0669]	<0.001	<0.001†	0.676		
	SNR	+0.019	+0.00235	[-0.00739, +0.01209]	0.637	0.825	0.001		
**Insula**<br>30.9 ± 5.6; R^2_{adj} = 0.649	Age (linear)	-0.358	-0.103	[-0.128, -0.078]	<0.001	<0.001†	0.267	111.8	Linear ↓
	Age^2	+0.042	+0.00077	[-0.00082, +0.00236]	0.343	0.774	0.005		
	Sex	-0.013	+0.15	[-0.921, +1.219]	0.786	0.829	<0.001		
	ICV	+0.711	+0.0276	[+0.0237, +0.0314]	<0.001	<0.001†	0.522		
	SNR	-0.012	-0.00087	[-0.00698, +0.00523]	0.780	0.825	<0.001		

ICV = intracranial volume; SNR = signal-to-noise ratio; p_{FDR} = Benjamini–Hochberg adjusted p-value, with correction applied separately within each predictor family ($m = 19$); * $p_{FDR} < 0.05$; ** $p_{FDR} < 0.01$; † $p_{FDR} < 0.001$; pR^2 = semi-partial R^2 (unique variance explained by each predictor, partialling out all others); R^2_{adj} shown in the region label cell; 95% CI constructed as $\beta \pm 1.96 \times SE$ (asymptotic); ‡ vertex age outside the observed range (16–82 yr), so the fitted quadratic is interpreted as effectively linear; ◇ quadratic association.
